# Tunable full-color dual-state (solution and solid) emission of push–pull molecules containing the 1-pyrindane moiety

**DOI:** 10.3762/bjoc.20.251

**Published:** 2024-11-19

**Authors:** Anastasia I Ershova, Sergey V Fedoseev, Konstantin V Lipin, Mikhail Yu Ievlev, Oleg E Nasakin, Oleg V Ershov

**Affiliations:** 1 Chuvash State University named after I.N. Ulyanov, Moskovsky pr., 15, Cheboksary, Russia

**Keywords:** dual-state emission, full-color emission, nitriles, push–pull molecules, pyrindane, stilbazole

## Abstract

A facile method for the synthesis of arylidene derivatives of pyrindane – (*E*)-7-arylmethylene-2-chloro-6,7-dihydro-5*H*-cyclopenta[*b*]pyridine-3,4-dicarbonitriles – was developed. Tunable full-color emission was achieved for the synthesized push–pull molecules, solely by changing donor groups while keeping both the conjugated system and acceptor part of the molecule unchanged. This represents a rare approach for the design of such fluorophores. Arylidene derivatives of pyrindane were found to be efficient fluorescent dyes showing a moderate to high emission quantum yield. The push–pull molecules were also characterized by a dual-state emission (in solution and in the solid state). Emission maxima ranged from 469 to 721 nm in solution depending on the solvent and type of donor substituent, and from 493 to 767 nm in the solid state. For the arylidene derivative of pyrindane with a dimethylamino group, it was shown that fluorescence can be changed by the action of an acid both in solution and in the solid state.

## Introduction

Over the past decades, heteroaromatic push–pull molecules have attracted great attention due to their widespread use in materials chemistry. This type of chromophores is of particular interest in the fields of organic electronics, photonics, and optoelectronics due to their unique optical and electronic properties [1–13]. Among heteroaromatic push–pull molecules, stilbazole derivatives (pyridostilbenes, azastilbenes, styrylpyridines or azinylarylethenes) are an important class. Uniquely, stilbazole provides a universal framework (exclusive matrix) for the design of donor–π–acceptor (D–π–A) molecules [14–15]. It has a branched π-conjugated system, in which the aromatic ring acts as a donor and pyridine as an acceptor. The introduction of additional substituents to stilbazole makes it possible to change the optical properties of this molecular framework within a wide range [16–19]. This approach has found many applications in the synthesis of compounds that are used in various optical materials [14,20–33]. For example, organic π-systems whose main structural unit is stilbazole are used as active compounds in organic light-emitting diodes (OLEDs) [20], dye-sensitized solar cells (DSSCs) [21], nonlinear optics (NLO) materials [22–23], positron emission tomography (PET) imaging [24], fluorescent probes and labels [25–27] detecting H_2_S in foodstuff, water, and living cells [28], Fe^3+^ ions [29], Hg^2+^ ions [30], and cyanide anions [31], for acid–base vapor sensing [32], and as candidate material for photonics devices, optical switches, and optical power limiting applications [33].

Materials with tunable full-color emission based on small organic molecules have attracted attention due to their great potential for applications [34–44]. These compounds provide unique benefits due to their flexibility, high efficiency, and versatility, making them essential for modern high-tech applications. Despite the wide variety of known push–pull molecules, the number of fluorescent cores with synthetic potential for tuning the emission wavelength to achieve a full-emission spectrum is limited. Typically, full-color fluorescence of organic molecules is achieved by extending π-conjugated systems or by introducing combinations of donor and acceptor groups, which changes the electronic properties and consequently the emission spectra [35–44]. This approach is synthetically challenging since it requires optimization of the reaction conditions for each modification step of the conjugated system. In this work, full-color fluorescence has been achieved solely by changing the donor groups, while the conjugated system and the multiacceptor part of the molecule were left unchanged, which is a rare approach for such fluorophores.

Another rare phenomenon for push–pull molecules is dual-state emission (DSE) [45–47]. At the same time, the scope of applications of fluorophores exhibiting DSE is much wider. This is due to the fact that DSE molecules, after absorbing energy, are able to emit in two different states (solution and solid state). This makes them more versatile and allows them to be used for the creation of fluorescent materials with different characteristics. Molecules exhibiting DSE are required to have certain structural features. First, they often contain donor and acceptor groups arranged in a specific sequence, such as in D–π–A chromophores. This creates the conditions for efficient intramolecular charge transfer (ICT), which plays a key role in the DSE phenomenon. Also, additional substituents can affect the geometry and conformation of the molecule, which may be important for the manifestation of the DSE phenomenon [45–46].

**Figure 1:**
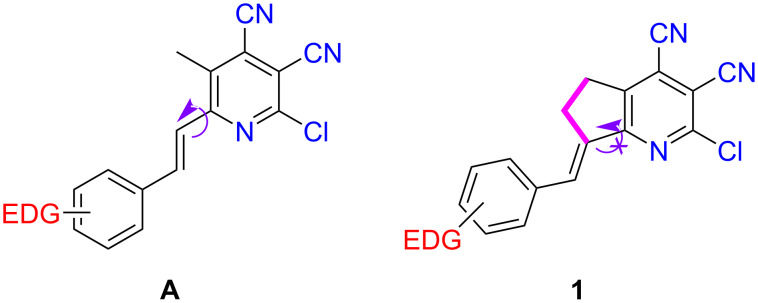
Structure of previously synthesized stilbazoles **А** and arylidene derivatives of pyrindane **1** reported herein.

Previously, we have reported the synthesis of stilbazoles **A** (Figure 1) [17]. In the present work, we developed a method for the synthesis of a rare class of compounds: arylidene derivatives of pyrindane **1** with conformational rigidity along the C–C bond between the heterocycle and ethene bridge due to the fused cyclic fragment. As a consequence of the additional ring, the fluorescence efficiency increased. At the same time, solid-state emission was observed due to the steric hindrance, which prevented intermolecular interactions in the nonplanar pyrindanes **1**. The obtained compounds **1**, having a 2-chloropyridine-3,4-dicarbonitrile moiety, contained easily modifiable functional groups [48–55]. This qualified compounds **1** as promising building blocks for diversity-oriented synthesis [56–57] and for the facile preparation of molecular libraries with an emphasis on skeletal diversity for the development of new push–pull molecules.

## Results and Discussion

### Synthesis and structure determination

**Scheme 1:**
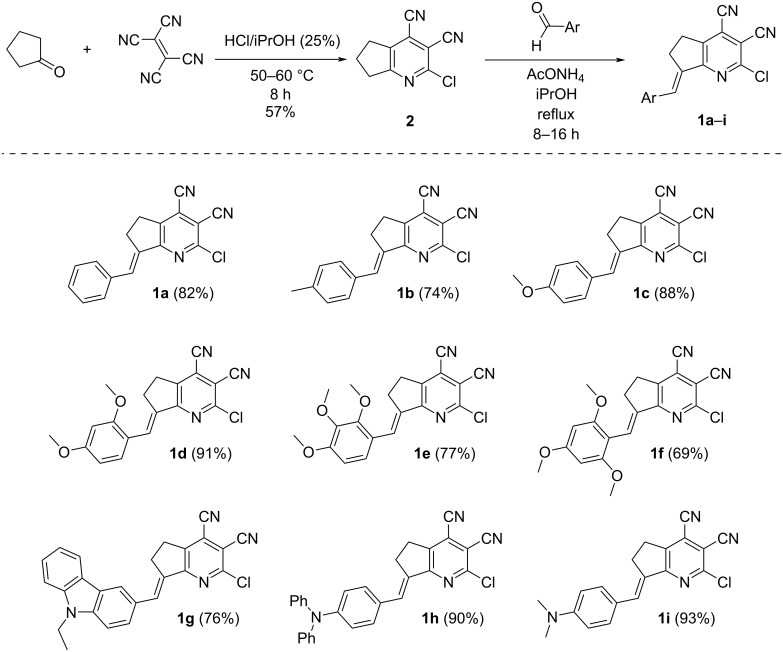
Synthesis of donor–acceptor 1-pyrindane derivatives **1**.

A two-step procedure was used to obtain the target compounds (Scheme 1). Cyclopenta[*b*]pyridine derivative **2** [58] was prepared in the first step via three-component reaction between tetracyanoethylene, cyclopentanone, and hydrogen chloride. Then, multiacceptor compound **2** was involved in the condensation with aromatic aldehydes bearing electron-donor groups. As a result, a series of new push–pull molecules containing various numbers of substituents at the donor site, which differed in their electron-donating strength, was obtained.

**Figure 2:**
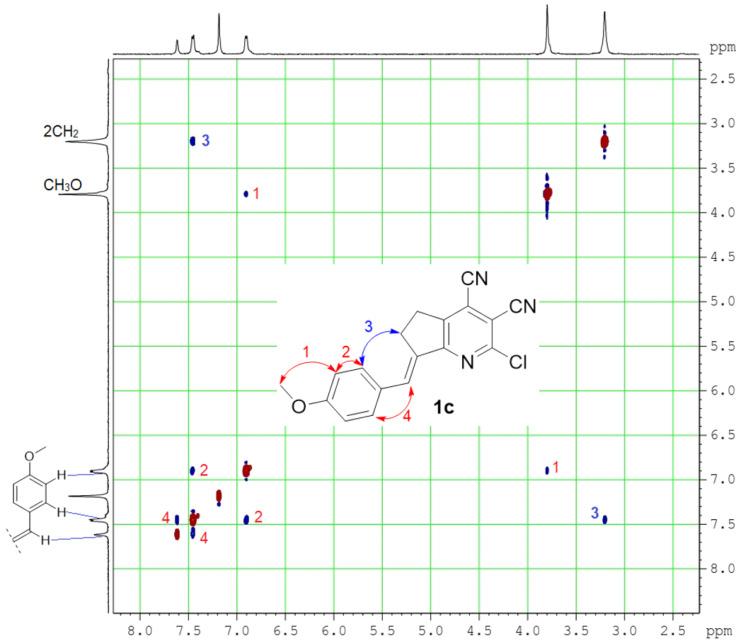
^1^H,^1^H-NOESY spectrum of compound **1c** in DMSO-*d*_6_.

It was found that the reaction of pyridine **2** with aromatic aldehydes occurred with complete stereoselectivity – only the *E-*isomer was obtained as the reaction product. The configuration of the double bond was confirmed using ^1^H,^1^H-NOESY spectroscopy. As shown in Figure 2, a correlation between protons of the allyl moiety and the aryl substituent evidenced their spatial proximity in molecule **1c**. The absence of a correlation between allyl and vinyl protons additionally supported the *E-*configuration.

### Spectral–luminescence properties

Compounds **1** form colored crystals, from pale-yellow (i.e., **1a**) to almost black (i.e., **1h**). They are soluble and luminescent in most common organic solvents. Solvatochromism of stilbazole **1c**, containing a *para*-methoxy group, was studied first (see Table S1 and Figure S1, Supporting Information File 1). The electronic absorption spectra were characterized by a pronounced maximum in the visible region centered at 431–448 nm. Emission maxima of compound **1c** were more significantly affected by the change of polarity and ranged from 475 nm (blue-green) in tetrachloromethane (CTC) to 588 nm (orange) in formic acid. Therefore, it was found that compound **1c** was characterized by a large Stokes shift upon increasing the solvent polarity, which reached 150 nm (5824 cm^−1^) in formic acid. This was associated with the bathochromic shift of the emission band, indicating that the more polar singlet excited state (*S*_1_) was much better stabilized by polar solvents than the ground state (*S*_0_). The highest fluorescence quantum yield of about 87% was observed in toluene.

**Figure 3:**
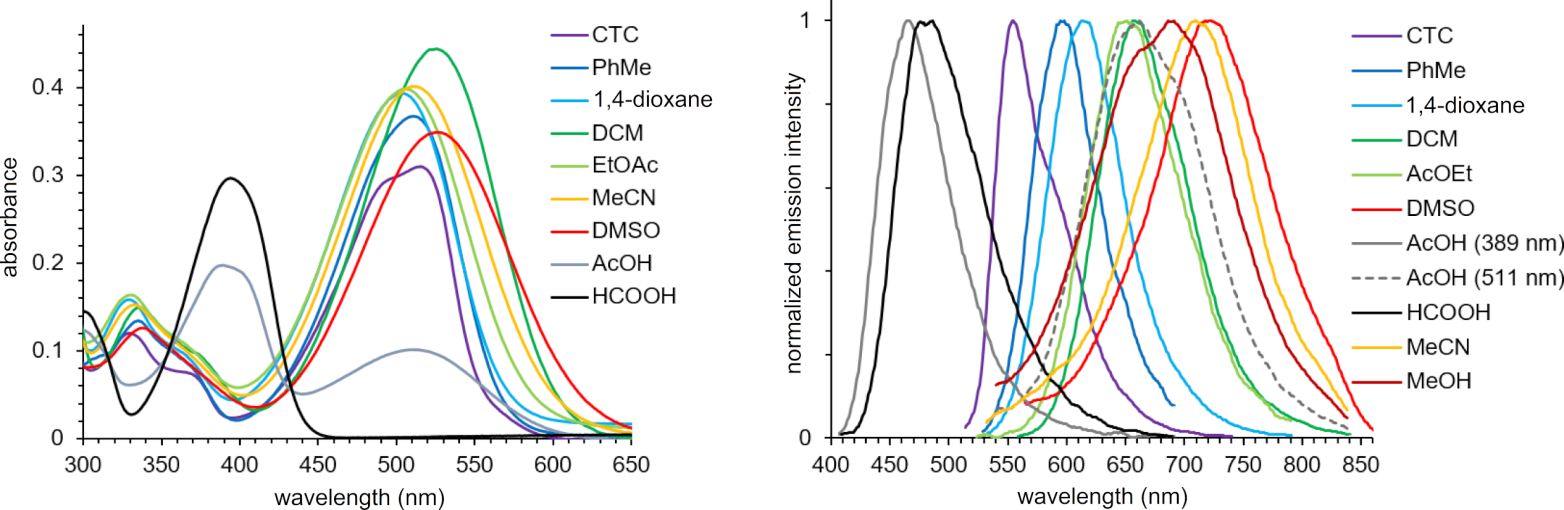
Absorption (left) and normalized emission spectra (right) of compound **1i** in various solvents (*c* = 10^−5^ M).

**Scheme 2:**
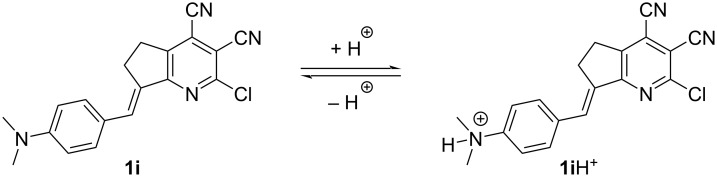
Plausible equilibrium of compounds **1i** and **1i**H^+^ in acidic solution.

Then, the solvatochromic properties of stilbazole **1i**, bearing a stronger electron-donating dimethylamino group, were studied (Table 1 and Figure 3). It was found that in most solvents, compound **1i** was characterized by a single pronounced absorption maximum in the range of 503–525 nm that red-shifted upon increasing the solvent polarity. In formic acid, due to the protonation of the dimethylamino group, a strong blue shift occurred down to 394 nm. The only exception was a solution of **1i** in acetic acid, where two peaks were observed. Apparently, the weaker acetic acid caused just a partial protonation of the amino group, and the equilibrium shown in Scheme 2 was observed. This was evidenced by two observed absorption maxima: the first almost coincided with the corresponding maximum of the solution in formic acid, and the second one was in the same region as with other aprotic solvents.

**Table 1:** Solvatochromic properties of compound **1i**.

solvent	λ_abs_, nm^a^	ε, M^−1^⋅cm^−1^	λ_em_, nm^b^	Stokes shift		Φ_em_, %^c^

				nm	cm^−1^	

CCl_4_ (CTC)	515	31000	554	39	1367	73.9
PhMe	510	36700	598	88	2885	49.5
1,4-dioxane	503	39400	614	111	3594	27.4
DCM	526	44400	656	130	3768	4.9
AcOEt	505	39900	651	146	4441	3.0
MeCN	510	40200	710	200	5523	0.7
DMSO	525	34900	721	196	5178	0.9
AcOH	389 511	19700 10100	467 662	78 151	4294 4464	24.6^d^ 1.8
HCOOH	394	28200	486	92	4805	30.9^d^
MeOH	510	—^e^	691	181	5136	0.1

^a^Absorption maxima were recorded in solution (*c* = 10^−5^ M). ^b^Emission maxima were recorded in solution (*c* = 10^−5^ M, absorption maxima were used for excitation. ^c^Relative emission quantum yield was estimated using a solution of rhodamine 6G in ethanol (Φ_em_ = 0.95 at 450 nm). ^d^Relative emission quantum yield was estimated using a solution of 7-hydroxy-4-methylcoumarin in phosphate buffer at pH 10 (Φ_em_ = 0.7 at 330 nm). ^e^Poorly soluble sample.

**Figure 4:**
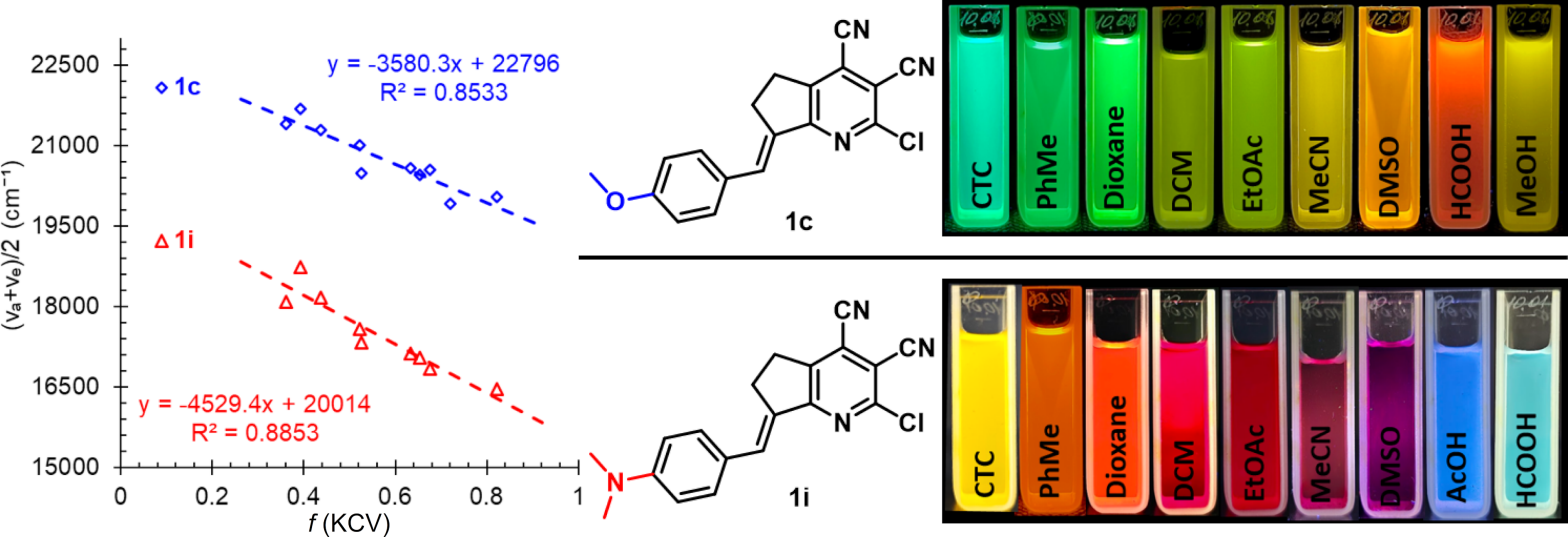
Solvatochromic behavior of compounds **1c** and **1i**: plots of arithmetic mean of emission/absorption wavenumbers vs Kawsk–Chamma–Viallet polarity function (left) and photos of fluorescent solutions in various solvents taken under a 365 nm UV lamp (right).

The emission maxima of stilbazole **1i** were within a very wide range of 264 nm and covered almost the entire visible region of the spectrum (from blue to red, Figure 3B and Figure 4). Acidic solutions were the most blue-shifted due to the formation of the protonated form **1i**H^+^ (Scheme 2). Two emission maxima were observed in acetic acid and associated with the corresponding absorption maxima. The first, located at 467 nm (excitation at 389 nm) was assigned to the formed **1i**H^+^ cation. This band showed a blue shift of 19 nm relative to formic acid due to the lower polarity of acetic acid. At the same time, the second band was assigned to the molecular form **1i** (Scheme 2) and observed at 662 nm (excitation at 511 nm), in the region of solvents with medium polarity. Protonation of the dimethylamino group was additionally confirmed by titration of pyrindane **1i** in toluene using trifluoroacetic acid (see Figure S2, Supporting Information File 1). According to the data obtained, an increasing amount of acid caused a blue shift of the maximum at 511 nm, and a new maximum in the region of 380–400 nm appeared in the absorption spectra. The intensity of the short-wavelength band also increased upon addition of trifluoroacetic acid. At the same time, a second band centered at 440 nm also appeared in the emission spectra. Additional evidence for protonation of the dimethylamino group in **1i**H^+^ (Scheme 2), rather than the pyridine fragment, was the solvatochromic behavior of compound **1c** (see Table S1, Supporting Information File 1). The solutions in AcOH and HCOOH did not show a strong blue shift since protonation did not occur. In these solvents, a classical pattern for the bathochromic emission shift was observed upon increasing the solvent polarity.

Generally, both stilbazoles **1c** and **1i** were characterized by solvatochromic behavior typical for molecules showing an ICT. A significant long-wavelength shift of the emission band was observed upon increasing the solvent polarity from carbon tetrachloride to DMSO, and the strongest fluorescence was registered in nonpolar medium (Φ_em_ = 87.5 % for compound **1c** in toluene and Φ_em_ = 73.9% for compound **1i** in CCl_4_). Slopes of the Lippert–Mataga plots (Figures S3 and S4, Supporting Information File 1) and the Kawski–Chamma–Viallet plots [59–60] (Figure 4, see Supporting Information File 1 for details) showed good linearity. This also indicated that the excited-state dipole moment of the molecules was much higher than that in the ground state. This phenomenon was even more pronounced for compound **1i** than for **1c** due to presence of the stronger electron-donating group.

**Figure 5:**
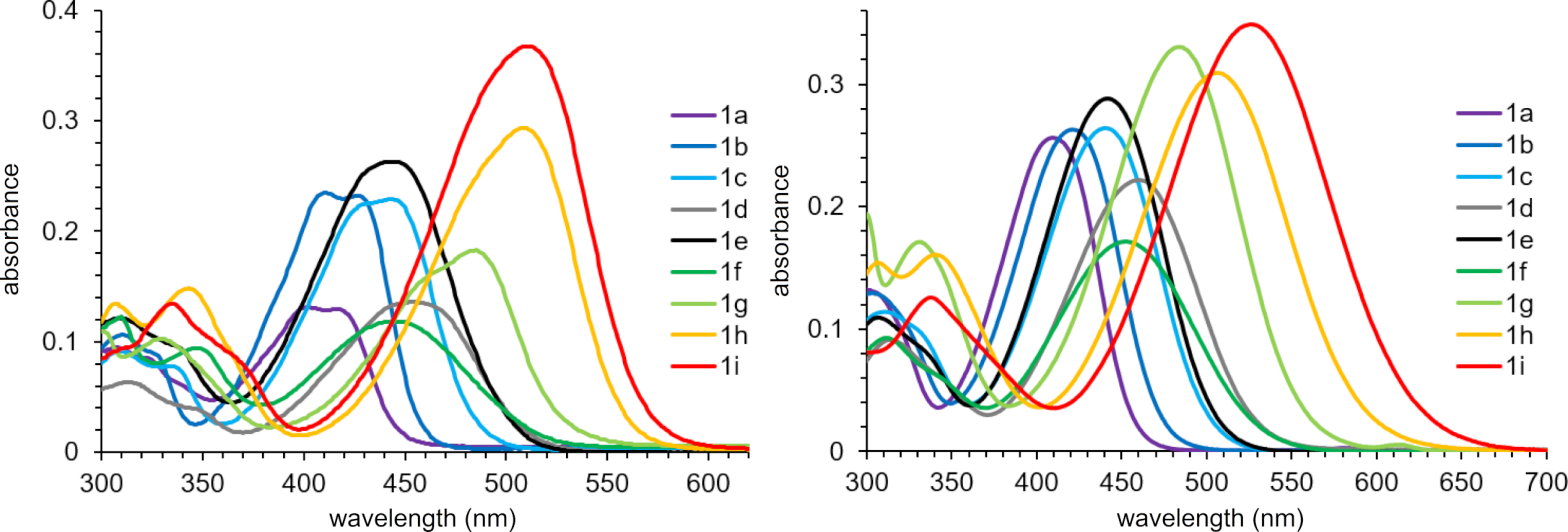
Absorption spectra of compounds **1a**–**i** in toluene (left) and DMSO (right, *c* = 10^−5^ M).

Then, the substituent effects on the spectral properties of stilbazoles **1a**–**i** were studied in two different solvents: nonpolar toluene (Table 2) and highly polar DMSO (Table 3). The absorption maxima of compounds **1a**–**i** were in the range of 402–510 nm (Figure 5, left). The most blue-shifted absorbance was observed for stilbazole **1a**, bearing no conjugated donor groups. Depending on their number and donor strength, the introduction of electron-donating substituents led to a bathochromic shift. The only exceptions were compounds **1e** and **1f**, containing three methoxy groups. In these cases, a blue shift of the absorption band in comparison to the disubstituted derivative **1d** was observed, which was apparently caused by a partial planarity violation due to steric hindrance. In DMSO, the absorption maxima of compounds **1a**–**i** were bathochromically shifted to 409–525 nm (Figure 5, right) and showed similar behavior to that described above.

**Table 2:** Photophysical properties of stilbazoles **1** in toluene.

compound	λ_abs_, nm^a^	ε, M^−1^⋅cm^−1^	λ_em_, nm^b^	Stokes shift		Φ_em_, %^c^

				nm	cm^−1^	

**1a**	402	13100	459	57	3089	32.9^d^
**1b**	411	23500	470	59	3054	12.2^d^
**1c**	443	22900	500	57	2573	87.5
**1d**	454	13600	520	66	2796	35.8
**1e**	444	26300	511	68	2953	43.2
**1f**	444	11800	531	87	3690	7.7
**1g**	485	18300	544	59	2236	53.1
**1h**	509	29400	582	73	2464	55.2
**1i**	510	36700	598	88	2885	49.5

^a^Absorption maxima were recorded in solution (*c* = 10^−5^ M). ^b^Emission maxima were recorded in solution (*c* = 10^−5^ M, absorption maxima were used for excitation). ^c^Relative emission quantum yield was estimated using a solution of rhodamine 6G in ethanol (Φ_em_ = 0.95 at 450 nm). ^d^Relative emission quantum yield was estimated using a solution of fluorescein in a 0.01 M KOH solution in ethanol (Φ_em_ = 0.97 at 425 nm).

**Table 3:** Photophysical properties of stilbazoles **1** in DMSO.

compound	λ_abs_, nm^a^	ε, M^−1^⋅cm^−1^	λ_em_, nm^b^	Stokes shift		Φ_em_, %^c^

				nm	cm^−1^	

**1a**	409	25600	506	97	4687	12.3^d^
**1b**	419	26300	528	109	4927	53.4^d^
**1c**	439	26400	578	139	5478	48.4
**1d**	460	22100	602	142	5128	20.5
**1e**	442	28800	596	154	5846	2.0
**1f**	453	17100	609	156	5655	5.1
**1g**	483	33000	649	166	5296	1.5
**1h**	505	31000	712	207	5757	0.2
**1i**	525	34900	721	196	5178	0.6

^a^Absorption maxima were recorded in solution (*c* = 10^−5^ M). ^b^Emission maxima were recorded in solution (*c* = 10^−5^ M, absorption maxima were used for excitation. ^c^Relative emission quantum yield was estimated using a solution of rhodamine 6G in ethanol (Φ_em_ = 0.95 at 450 nm). ^d^Relative emission quantum yield was estimated using a solution of fluorescein in a 0.01 M KOH solution in ethanol (Φ_em_ = 0.97 at 425 nm).

**Figure 6:**
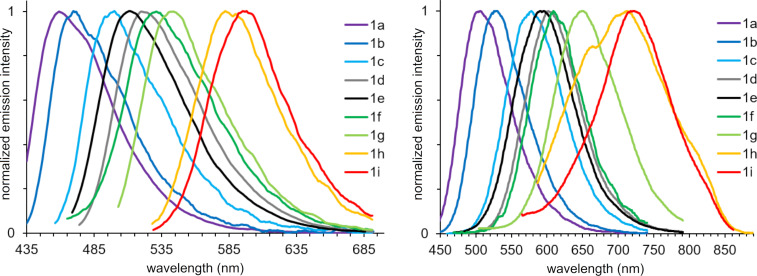
Normalized emission spectra of compounds **1a**–**i** in toluene (left) and DMSO (right, *c* = 10^−5^ M).

**Figure 7:**
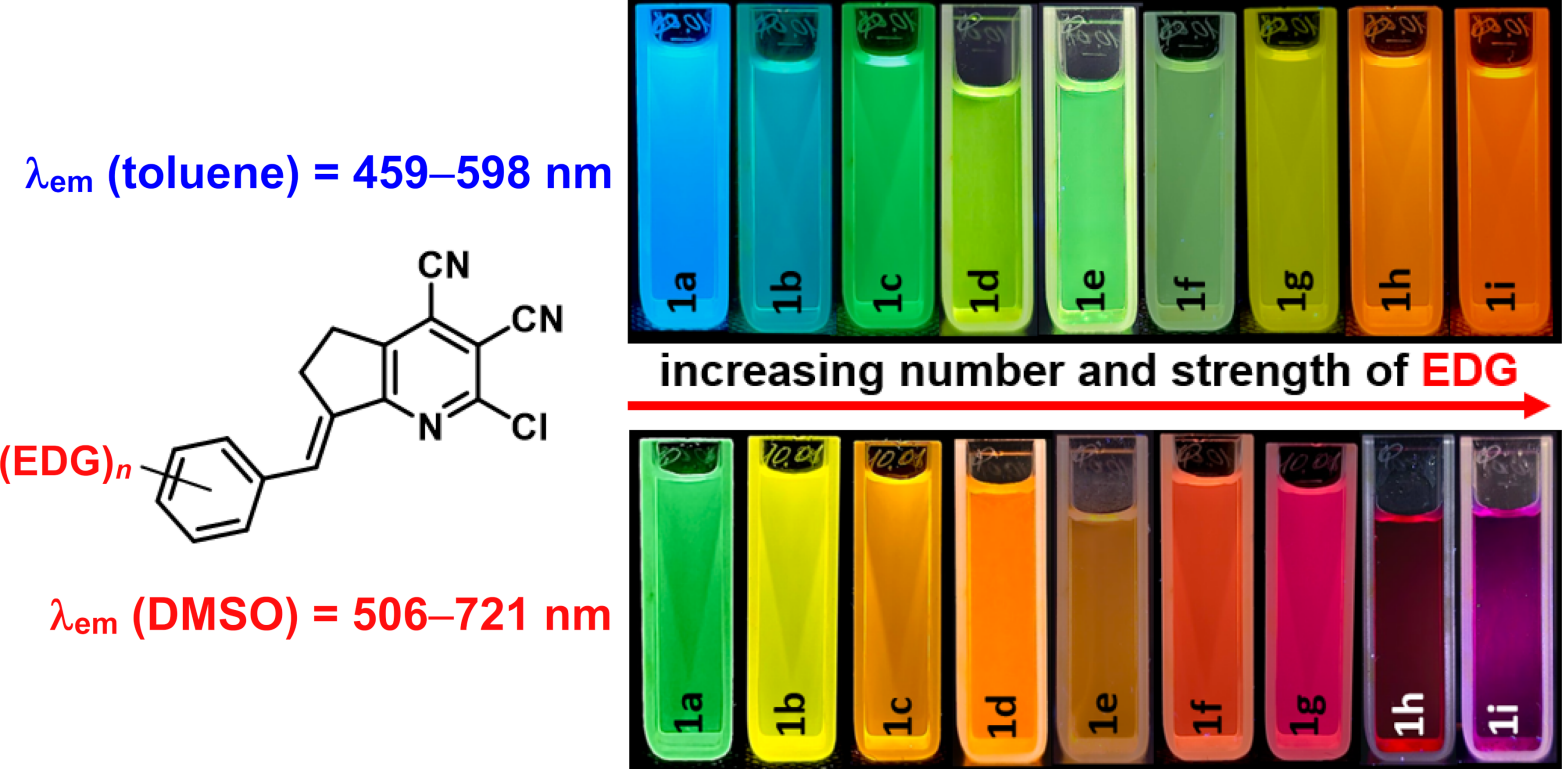
Photos of fluorescent solutions of compounds **1a**–**i** in toluene (top) and DMSO (bottom) taken under a 365 nm UV lamp.

The photoluminescence spectra of stilbazoles **1a**–**i** in toluene were characterized by a maximum in the range of 459–598 nm (Figure 6, left), associated with an emission color from blue to orange (Figure 7, top). The most blue-shifted emission was observed for stilbazole **1a** without additional substituents. The introduction of an electron-donating group led to a red shift of the emission in accordance with increasing donor strength and number of substituents. Stilbazoles **1** in toluene were characterized by a high fluorescence quantum yield, reaching 87.5% for the *para-*methoxy-substituted derivative **1c**. Emission maxima in DMSO were found to be in the range of 506–721 nm (Figure 6, right), associated with a fluorescence color from green to red (Figure 7, bottom). The highest fluorescence efficiency of 53.4% was observed for the *para-*methyl derivative **1b**. Solutions of stilbazoles **1** in DMSO were also characterized by large Stokes shift values, reaching 207 nm (5846 cm^−1^) and showing nonradiative loss of excitation energy.

**Figure 8:**
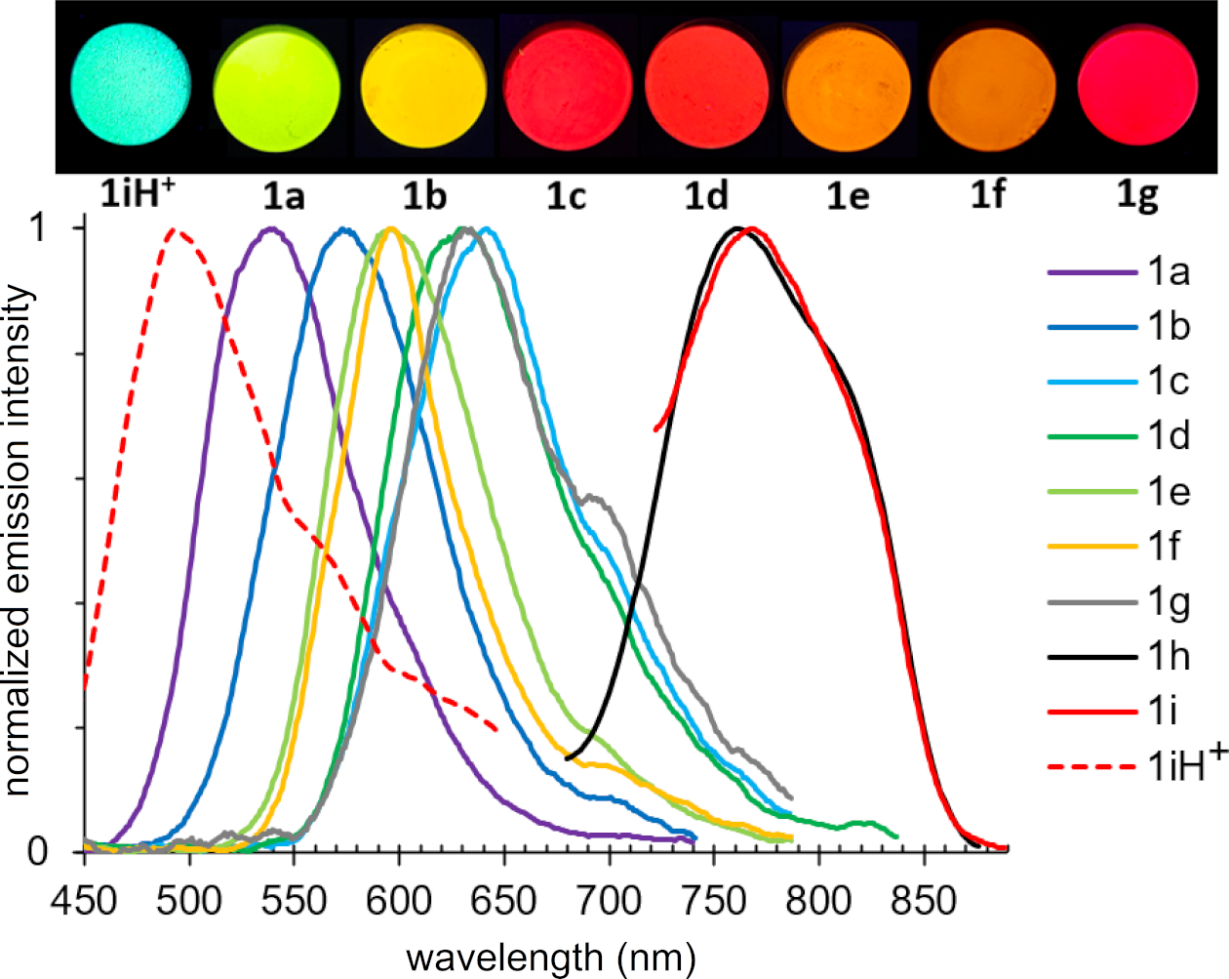
Normalized solid-state emission spectra of compounds **1a**–**i** (bottom) and photos of powders taken under a 365 nm UV lamp (top).

It should be noted that stilbazoles **1**, in contrast to related compounds **A** (Figure 1), were characterized by solid-state emission (Table 4 and Figure 8). This was apparently caused by the presence of the dimethylene bridge, preventing intermolecular interactions. Emission maxima of compounds **1** ranged from 540–767 nm, namely from the green to the near-infrared region of the spectrum. The highest intensity was observed for stilbazole **1a**, bearing no donor groups. The emission intensity decreased upon increasing the donor strength of the substituent. As shown in Scheme 2 and according to the fluorescence spectra recorded in acidic solutions, compound **1i** could form the salt **1i**H^+^. Therefore, the effect of acid vapors on the solid-state emission was studied. It was found that pyrindane **1i** was sensitive to formic and trifluoroacetic acid vapors. As a result of protonation, a significant blue shift of the emission maximum from 762 nm down to 493 nm was observed.

**Table 4:** Solid-state photoluminescence of stilbazoles **1**.

compound	λ_em_, nm	emission intensity, a.u.^a^

**1a**	540	798
**1b**	573	391
**1c**	641	139
**1d**	629	127
**1e**	596	202
**1f**	596	142
**1g**	631	77
**1h**	795	31
**1i**	762	12
**1i**H^+^	493	322

^a^Emission intensity is given in arbitrary units (a.u.) of the Cary Eclipse fluorescence spectrometer, see Supporting Information File 1 for details.

## Conclusion

A method for the synthesis of new push–pull stilbazoles of the type D–π–A was developed. The obtained compounds represent a rare class of benzylidene derivatives of 1-pyrindane. They were characterized by an unusual type of photoluminescence in two states (dual-state emission), namely in solution and in the solid state. Emission in solution was in the range of 469–721 nm, depending on the solvent, number, and type of substituent and covered almost the entire visible spectrum. In the solid state, the emission ranged from 493–767 nm. It was found that the presence of a dimethylene bridge in arylidene derivatives of pyrindane **1** led to an increase in the emission quantum yield and caused an appearance of solid-state photoluminescence, in contrast to the previously synthesized analogues (stilbazoles **A**, Figure 1) without such a bridge (Tables S3 and S4, Supporting Information File 1). Moreover, it was found that the emission band could be adjusted by about 200 nm in solution and by 270 nm in the solid state through directed protonation of the dimethylamino group.

## Supporting Information

File 1 Synthetic procedure and compound characterization data, solvatochromic studies for compound **1с**, titration data, and ^1^H and ^13^C NMR spectra for compounds **1a**–**i**.

## Data Availability

All data that supports the findings of this study is available in the published article and/or the supporting information of this article.

## References

[1] Bureš F (2014). RSC Adv.

[2] Klikar M, Solanke P, Tydlitát J, Bureš F (2016). Chem Rec.

[3] Wang J, Gadenne V, Patrone L, Raimundo J-M (2024). Molecules.

[4] He G S, Tan L-S, Zheng Q, Prasad P N (2008). Chem Rev.

[5] Allard S, Forster M, Souharce B, Thiem H, Scherf U (2008). Angew Chem, Int Ed.

[6] Ohmori Y (2010). Laser Photonics Rev.

[7] Jaswal S, Kumar J (2020). Mater Today: Proc.

[8] Wang L, Zhu W (2024). Adv Sci.

[9] Wu Y, Zhu W (2013). Chem Soc Rev.

[10] Sil A, Ghosh U, Dolai S, Manna S, Maity A, Patra S K (2022). Mater Adv.

[11] Huang T, Jiang W, Duan L (2018). J Mater Chem C.

[12] Da Lama A, Sestelo J P, Valencia L, Esteban-Gómez D, Sarandeses L A, Martínez M M (2022). Dyes Pigm.

[13] Stanitska M, Volyniuk D, Minaev B, Agren H, Grazulevicius J V (2024). J Mater Chem C.

[14] Lipunova G N, Nosova E V, Trashakhova T V, Charushin V N (2011). Russ Chem Rev.

[15] Sorokin S P, Ershov O V (2022). Chem Heterocycl Compd.

[16] Sorokin S P, Ievlev M Y, Ershov O V (2023). Dyes Pigm.

[17] Ershova A I, Fedoseev S V, Blinov S A, Ievlev M Y, Lipin K V, Ershov O V (2023). Org Biomol Chem.

[18] Sorokin S P, Ievlev M Y, Ershov O V (2024). Org Biomol Chem.

[19] Cao C, Zeng Z, Cao C (2022). J Phys Org Chem.

[20] Choi H-J, Song M-G, Sim Y-H, Bae H-K, Kim J-W, Park L S (2010). Mol Cryst Liq Cryst.

[21] Risi G, Devereux M, Prescimone A, Housecroft C E, Constable E C (2023). RSC Adv.

[22] Poornima L, Babu R S, Kalainathan S (2023). J Mol Struct.

[23] Wang T, Ma J, Xu K, Chen R, Cao L, Teng B (2022). Cryst Growth Des.

[24] Beuché S, Peyronneau M-A, Jego B, Denis C, Bourbon P, Chauvière C, Santerre C, Truillet C, Kuhnast B, Caillé F (2023). J Med Chem.

[25] Xiong Q, Zhao K, Cheng Y, He C, Lai Y, Shi M, Ming X, Jin F, Tao D, Liao R (2023). Spectrochim Acta, Part A.

[26] Singh D, Shewale D J, Sengupta A, Soppina V, Kanvah S (2022). Org Biomol Chem.

[27] Luo Y, Yu Q-Q, Gao J-J, Lang X-X, Li H-Y, Yu X-F, Qi X-Y, Wang M-Q (2021). Bioorg Med Chem Lett.

[28] Xie L, Wang R, Fan C, Tu Y, Liu G, Pu S (2023). Food Chem.

[29] Feng X, Li Y, He X, Liu H, Zhao Z, Kwok R T K, Elsegood M R J, Lam J W Y, Tang B Z (2018). Adv Funct Mater.

[30] Zhou H, Sun L, Chen W, Tian G, Chen Y, Li Y, Su J (2016). Tetrahedron.

[31] Liang M, Wang K, Guan R, Liu Z, Cao D, Wu Q, Shan Y, Xu Y (2016). Spectrochim Acta, Part A.

[32] Ma C, He J, Wu Y, Li J, Chen J, Zhang Y, Mo J, Xie H, Chi Z, Li Y (2023). Luminescence.

[33] Senthil K, Kalainathan S, Kumar A R, Aravindan P G (2014). RSC Adv.

[34] Zhou C, Wang M, Guo W, Ye G, Wang Y, Yang Y, Xia G, Wang H (2023). Dyes Pigm.

[35] Xu Z, Liao Q, Shi X, Li H, Zhang H, Fu H (2013). J Mater Chem B.

[36] Kim E, Koh M, Ryu J, Park S B (2008). J Am Chem Soc.

[37] Radhakrishnan R, Sinu B B, Anilkumar V, Sreejalekshmi K G (2020). Dyes Pigm.

[38] Zhu P, Yang Y, Li H, Wang J, Li S (2024). Chin Chem Lett.

[39] Zhu Y, Liao K, Li Y, Zhang W, Song B, Hao X-Q, Zhu X (2024). Dyes Pigm.

[40] Zhang X, Wang D, Shen H, Wang S, Zhou Y, Lei Y, Gao W, Liu M, Huang X, Wu H (2021). Org Chem Front.

[41] Chen S-H, Cao X-Y, Hu P-T, Jiang K, Liang Y-T, Xu B-J, Li Z-H, Wang Z-Y (2023). Mater Adv.

[42] Chen Z, Qin H, Yin Y, Deng D-d, Qin S-Y, Li N, Wang K, Sun Y (2023). Chem – Eur J.

[43] Wen W, Shi Z-F, Cao X-P, Xu N-S (2016). Dyes Pigm.

[44] Ruan B, Kang X, Guo B, Deng D-d, Tian J-j, He K, Wang X-Y, Pu S, Chen Z (2024). J Mol Struct.

[45] Belmonte-Vázquez J L, Amador-Sánchez Y A, Rodríguez-Cortés L A, Rodríguez-Molina B (2021). Chem Mater.

[46] Xia G, Si L, Wang H (2023). Mater Today Chem.

[47] Stoerkler T, Pariat T, Laurent A D, Jacquemin D, Ulrich G, Massue J (2022). Molecules.

[48] Vachova L, Machacek M, Kučera R, Demuth J, Cermak P, Kopecky K, Miletin M, Jedlickova A, Simunek T, Novakova V (2015). Org Biomol Chem.

[49] Ershova A I, Ievlev M Y, Maksimova V N, Ershov O V (2022). Russ J Gen Chem.

[50] Fedoseev S V, Belikov M Y, Lipin K V, Ershov O V, Tafeenko V A (2022). Synth Commun.

[51] Chunikhin S S, Ershov O V, Ievlev M Y, Belikov M Y, Tafeenko V A (2018). Dyes Pigm.

[52] Arafa W A A, Hussein M F (2020). Chin J Chem.

[53] Ershov O V, Shishlikova M A, Ievlev M Y, Belikov M Y, Maksimova V N (2019). Tetrahedron.

[54] Maximova V N, Naidenova A I, Ershov O V, Nasakin O E, Tafeenko V A (2017). Russ J Org Chem.

[55] Chunikhin S S, Ershov O V (2021). Russ J Org Chem.

[56] Lenci E, Trabocchi A (2022). Eur J Org Chem.

[57] Spring D R (2003). Org Biomol Chem.

[58] Ershov O V, Maksimova V N, Lipin K V, Belikov M Y, Ievlev M Y, Tafeenko V A, Nasakin O E (2015). Tetrahedron.

[59] Kawski A (2002). Z Naturforsch, A: Phys Sci.

[60] Chamma A, Viallet P (1970). C R Seances Acad Sci, Ser C.

