# Supplementary material for: Tunable full-color dual-state (solution and solid) emission of push–pull molecules containing the 1-pyrindane moiety
Source: Beilstein J Org Chem. 2024 Nov 19;20:3016–25. doi: 10.3762/bjoc.20.251 (PMC11590021; doi:10.3762/bjoc.20.251)
Supplement: File 1 — Synthetic procedure and compound characterization data, solvatochromic studies for compound 1с, titration data, and 1H and 13C NMR spectra for compounds 1a–i. [file Beilstein_J_Org_Chem-20-3016-s001.pdf]

BEILSTEIN JOURNAL OF ORGANIC CHEMISTRY

Supporting Information

for

Tunable full-color dual-state (solution and solid) emission of push–pull molecules containing the 1-pyrindane moiety

Anastasia I. Ershova, Sergey V. Fedoseev, Konstantin V. Lipin, Mikhail Yu. Ievlev, Oleg E. Nasakin and Oleg V. Ershov

Beilstein J. Org. Chem. doi:

Synthetic procedure and compound characterization data, solvatochromic studies for compound 1c, titration data, and ^{1}H and ^{13}C NMR spectra for compounds 1a–i

License and Terms: This is a supporting information file under the terms of the Creative Commons Attribution License (https://creativecommons.org/licenses/by/4.0). Please note that the reuse, redistribution and reproduction in particular requires that the author(s) and source are credited and that individual graphics may be subject to special legal provisions.
The license is subject to the *Beilstein Journal of Organic Chemistry* terms and conditions: (https://www.beilstein-journals.org/bjoc/terms)

CONTENTS

1. Experimental S1

2. Solvatochromic studies data S6

3. Titration experment data S7

4. Lippert–Mataga plots S7

5. Kawski–Chamma–Viallet's equation S8

6. Comparison of photo-physical properties of stilbazoles 1 and A S9

7. Copies of ^{1}H and ^{13}C NMR sprectra S11

8. References S23

1. Experimental

1.1. Materials and instrumentation

All reagents (cyclopentanone, tetracyanoethylene, aromatic aldehydes, ammonium acetate, 10%-solution of hydrogen chloride in propan-2-ol) are commercial products and were used without additional purification. All solvents (carbon tetrachloride, toluene, 1,4-dioxane, ethyl acetate, acetic acid, formic acid, dichloromethane, dimethyl sulfoxide, acetonitrile, methanol, propan-2-ol) used for solvatochromic behavior studies were of spectroscopic grade and used without further purification. Reaction progress and purity of the synthesized compounds were monitored by TLC method, spots were visualized by UV-irradiation (254 or 365 nm), iodine vapors and thermal decomposition. Melting points were determined on an OptiMelt MPA100 (USA) apparatus. Elemental analyses were performed on a FlashEA 1112 CHN-analyzer (USA). NMR spectra were measured on a DRX-500 spectrometer (Bruker, USA) in DMSO-d_6 or $CDCl_3$ solutions using TMS as an internal standard, working frequency was 500.13 MHz for ^1H and 125.76 MHz for ^{13}C NMR experiments. Mass spectra were recorded on a Shimadzu GCMS-QP2020 (Japan) (electron impact energy was 70 eV). Electronic absorption spectra were obtained on an Agilent Cary 60 UV–vis Spectrophotometer (USA) using standard 1 cm quartz cuvette. Photoluminescence spectra were registered on an Agilent Cary Eclipse fluorescence spectrometer (USA). Fluorescence quantum yields (Φ_{em}) were estimated by the creation of a calibration curve plotting the area of emission against absorbance for different concentrations of the sample using rhodamine 6G in ethanol (Φ_{em} 0.95 at 450 nm) [1], fluorescein in 0.01 M KOH in ethanol (Φ_{em} 97% at λ_{ex} 425 nm) [2] and 7-hydroxy-4-methylcoumarin in phosphate buffer at pH 10 (Φ_{em} 0.7 at 330 nm) [3] as standards. Solid state emission spectra of powdered samples were recorded at room temperature on an Agilent Cary Eclipse fluorescence spectrometer (USA) using a solid sample holder. For comparative purposes all experiments were carried out in the same conditions. The slits of the excitation and emission monochromators were set at 2.5 nm and 5 nm respectively, the photomultiplier voltage was set at 550 V, and wavelength of 350 nm was used for excitation.

1.2. Synthetic procedures

Synthesis of 2-chloro-6,7-dihydro-5*H*-cyclopenta[*b*]pyridine-3,4-dicarbonitrile (2). Cyclopentanone (0.84 g, 10 mmol) was added to a pre-heated up to 50 °C solution of propan-2-ol (10 ml) containing 10% (w/w) of hydrogen chloride followed by addition of tetracyanoethylene (10 mmol, 1.28 g). The reaction mixture was stirred at 50–60 °C for 5 h. Then the mixture was cooled down, the precipitated solid was filtered off, washed with water and propan-2-ol. An additional amount of compound **2** was isolated by dilution of the filtrate with water (50 mL). The precipitated solid was filtered off and crystallized from propan-2-ol.

General method for the preparation of 1-pyrindanes (1). 2-Chloro-6,7-dihydro-5*H*-cyclopenta[*b*]pyridine-3,4-dicarbonitrile (**2**, 0.2 g, 1 mmol), appropriate aromatic aldehyde (1 mmol) and ammonium acetate (0.08 g, 1 mmol) were added to propan-2-ol (5 ml). The reaction mixture was heated at reflux for 8–16 h (TLC controlled). Then the mixture was cooled down, the precipitated solid was filtered off and washed with propan-2-ol.

(*E*)-7-Benzylidene-2-chloro-6,7-dihydro-5*H*-cyclopenta[*b*]pyridine-3,4-dicarbonitrile (**1a**). Yellow crystals. Yield 82%. M.p. 233-234 °C (dec.). ^{1}H NMR (500 MHz, DMSO-d_6) δ 3.26–3.29 (m, >4H, $2CH_2$, HDO), 7.41 (t, *J* = 7.3 Hz, 1H, C_6H_5), 7.48 (t, *J* = 7.5 Hz, 2H, C_6H_5), 7.58 (s, 1H, CH=C), 7.66 (d, *J* = 7.6 Hz, 2H, C_6H_5). ^{11}H NMR (500 MHz, $CDCl_3$) δ 3.31 (s, 4H, $2CH_2$), 7.38-7.42 (m, 1H, C_6H_5), 7.46 (t, *J* = 7.5 Hz, 2H, C_6H_5), 7.56 (d, *J* = 7.7 Hz, 2H, C_6H_5), 7.73 (s, 1H, CH=C). ^{13}C NMR (126 MHz, DMSO-d_6) δ 26.9 (CH_2), 27.6 (CH_2), 106.8 (β-Py[C]–CN), 112.2 (CN), 113.1 (CN), 121.3 (γ-Py[C]–CN), 128.4 (2C, C_6H_5), 128.6 (1C, C_6H_5), 129.1 (CH=C), 129.4 (2C, C_6H_5), 135.1 (1C, C_6H_5), 137.8 (CH=C), 141.7 (β-Py[C]–CH_2), 151.1 (α-Py[C]–Cl), 165.4 (α-Py[C]–C). MS (EI): m/z = 290 [M^+-1H] (100), 290 [M^+ (^{35}Cl)] (63), 293 [M^+ (^{37}Cl)] (21). Anal. Calcd for $C_{17}H_{10}ClN_3$: C, 69.99; H, 3.46; N, 14.40. Found, C, 70.11; H, 3.42; N, 14.37.

(*E*)-2-Chloro-7-(4-methylbenzylidene)-6,7-dihydro-5*H*-cyclopenta[*b*]pyridine-3,4-dicarbonitrile (**1b**). Yellow crystals. Yield 74%. M.p. 244-245 °C (dec.). ^{1}H NMR (500 MHz, DMSO-d_6) δ 2.36 (s, 3H, CH_3), 3.19–3.26 (m, >4H, $2CH_2$, HDO), 7.28 (d, *J* = 8.0 Hz, 2H, C_6H_4), 7.51-7.58 (m, 3H, CH=C, C_6H_4). ^{1}H NMR (500 MHz, $CDCl_3$) δ 2.41 (s, 3H, CH_3), 3.29 (s, 4H, $2CH_2$), 7.25–7.28 (d, *J* = 7.8 Hz, >2H, C_6H_4, $CHCl_3$), 7.47 (d, *J* = 7.8 Hz, 2H, C_6H_4), 7.72 (s, 1H, CH=C). ^{13}C NMR (126 MHz, DMSO-d_6) δ 20.4 (CH_3), 26.8 (CH_2), 27.6 (CH_2), 106.4 (β-Py[C]–CN), 112.2 (CN), 113.1 (CN), 121.1 (γ-Py[C]–CN), 129.0 (2C, C_6H_4), 129.2 (CH=C), 129.4 (2C, C_6H_4), 132.4 (1C, C_6H_4), 136.8 (CH=C), 138.7 (Me[C]C_6H_4), 141.6 (β-Py[C]–CH_2), 151.1 (α-Py[C]–Cl), 165.6 (α-Py[C]–C). MS (EI): m/z = 305 [M^+ (^{35}Cl)] (100), 307 [M^+ (^{37}Cl)] (34). Anal. Calcd for $C_{18}H_{12}ClN_3$: C, 70.71; H, 3.96; N, 13.74. Found, C, 70.59; H, 4.00; N, 13.78.

(*E*)-2-Chloro-7-(4-methoxybenzylidene)-6,7-dihydro-5*H*-cyclopenta[*b*]pyridine-3,4-dicarbonitrile (**1c**). Orange crystals. Yield 88%. M.p. 269-270 °C (dec.). ^{1}H NMR (500 MHz, DMSO-d_6) δ 3.18–3.30 (m, >4H, $2CH_2$, HDO), 3.83 (s, 3H, OCH_3), 7.05 (d, *J* = 8.1 Hz, 2H, C_6H_4), 7.57 (s, 1H, CH=C), 7.64 (d, *J* = 8.0 Hz, 2H, C_6H_4). ^{1}H NMR (500 MHz, $CDCl_3$) δ 3.24–3.31 (m, 4H, $2CH_2$), 3.87 (s, 3H, OCH_3), 6.98 (d, *J* = 8.8 Hz, 2H, C_6H_4), 7.53 (d, *J* = 8.7 Hz, 2H, C_6H_4), 7.69 (s, 1H, CH=C). ^{13}C NMR (126 MHz, DMSO-d_6) δ 26.7 (CH_2), 27.5 (CH_2), 54.9 (OCH_3), 105.7 (β-Py[C]–CN), 112.1 (CN), 113.0 (CN), 114.1 (2C, C_6H_4), 120.7 (γ-Py[C]–CN), 127.8 (1C, C_6H_4), 129.2 (CH=C), 131.2 (2C, C_6H_4), 135.0 (CH=C), 141.4 (β-Py[C]–CH_2), 151.0 (α-Py[C]–Cl), 159.8 (MeO[C]C_6H_4), 165.7 (α-Py[C]–C). MS (EI): m/z = 321 [M^+ (^{35}Cl)] (100), 323 [M^+ (^{37}Cl)] (33). Anal. Calcd for $C_{18}H_{12}ClN_3O$: C, 67.19; H, 3.76; N, 13.06. Found, C, 67.25; H, 3.74; N, 13.01.

(*E*)-2-Chloro-7-(2,4-dimethoxybenzylidene)-6,7-dihydro-5*H*-cyclopenta[*b*]pyridine-3,4-dicarbonitrile (**1d**). Red crystals. Yield 91%. M.p. 311-313 °C (dec.). ^{1}H NMR (500 MHz, DMSO-d_6) δ 3.14–3.20 (m, 2H, CH_2), 3.21–3.25 (m, 2H, CH_2), 3.86 (s, 3H, OCH_3), 3.90 (s, 3H, OCH_3), 6.63–6.67 (m, 2H, C_6H_3), 7.55 (d, *J* = 8.4 Hz, 1H, C_6H_3), 7.88 (s, 1H, CH=C). ^{13}C NMR (126 MHz, DMSO-d_6) δ 26.7 (CH_2), 27.6 (CH_2), 55.1 (OCH_3), 55.6 (OCH_3), 98.4 (1C, C_6H_3), 105.4 (β-Py[C]–CN), 105.9 (1C, C_6H_3), 112.3 (CN), 113.2 (CN), 117.1 (1C, C_6H_3), 120.6 (γ-Py[C]–CN), 123.8 (1C, C_6H_3), 129.7 (CH=C), 134.6 (CH=C), 141.5 (β-Py[C]–CH_2), 151.1 (α-Py[C]–Cl), 159.4 (MeO[C]C_6H_3), 165.7 (α-Py[C]–C), 166.1 (MeO[C]C_6H_3). MS (EI): m/z = 351 [M^+ (^{35}Cl)] (100), 353 [M^+ (^{37}Cl)] (33). Anal. Calcd for $C_{19}H_{14}ClN_3O_2$: C, 64.87; H, 4.01; N, 11.94. Found, C, 64.98; H, 3.97; N, 11.90.

(*E*)-2-Chloro-7-(2,3,4-trimethoxybenzylidene)-6,7-dihydro-5*H*-cyclopenta[*b*]pyridine-3,4-dicarbonitrile (**1e**). Orange crystals. Yield 77%. M.p. 259-261°C (dec.). ^{1}H NMR (500 MHz, DMSO-d_6) δ 3.16–3.27 (m, >4H, $2CH_2$, HDO), 3.80 (s, 3H, OCH_3), 3.86 (s, 3H, OCH_3), 3.88 (s, 3H, OCH_3), 6.93 (d, *J* = 8.9 Hz, 1H, C_6H_2), 7.55 (d, *J* = 8.8 Hz, 1H, C_6H_2), 7.78 (s, 1H, CH=C). ^{1}H NMR (500 MHz, $CDCl_3$) δ 3.20–3.28 (m, 4H, $2CH_2$), 3.90 (s, 3H, OCH_3), 3.92 (s, 3H, OCH_3), 3.96 (s, 3H, OCH_3), 6.75 (d, *J* = 8.8 Hz, 1H, C_6H_2), 7.26–7.29 (m, >1H, C_6H_2, $CHCl_3$), 7.99 (s, 1H, CH=C). ^{13}C NMR (126 MHz, DMSO-d_6) δ 26.8 (CH_2), 27.6 (CH_2), 55.8 (OCH_3), 60.0 (OCH_3), 61.0 (OCH_3), 106.0 (β-Py[C]–CN), 108.1 (1C, C_6H_2), 112.3 (CN), 113.2 (CN), 120.9 (γ-Py[C]–CN), 121.9 (1C, C_6H_2), 123.5 (CH=C), 123.6 (1C, C_6H_2), 136.1 (CH=C), 141.6 (β-Py[C]–CH_2), 141.8 (MeO[C]C_6H_2), 151.1 (α-Py[C]–Cl), 152.5 (MeO[C]C_6H_2), 154.5 (MeO[C]C_6H_2), 165.8 (α-Py[C]–C). MS (EI): m/z = 381 [M^+ (^{35}Cl)] (100), 383 [M^+ (^{37}Cl)] (33). Anal. Calcd for $C_{20}H_{16}ClN_3O_3$: C, 62.92; H, 4.22; N, 11.01. Found, C, 63.05; H, 4.18; N, 10.96.

(*E*)-2-Chloro-7-(2,4,6-trimethoxybenzylidene)-6,7-dihydro-5*H*-cyclopenta[*b*]pyridine-3,4-dicarbonitrile (**1f**). Orange crystals. Yield 69%. M.p. 273-275 °C (dec.). ^{1}H NMR (500 MHz, DMSO-d_6) δ 2.75–2.80 (m, 2H, CH_2), 3.08–3.13 (m, 2H, CH_2) 3.82 (s, 6H, $2OCH_3$), 3.84 (s, 3H, OCH_3), 6.30 (s, 2H, C_6H_2), 7.51 (s, 1H, CH=C). ^{1}H NMR (500 MHz, $CDCl_3$) δ 2.86–2.91 (m, 2H, CH_2), 3.10–3.14 (m, 2H, CH_2), 3.85 (s, 6H, $2OCH_3$), 3.86 (s, 3H, OCH_3), 6.16 (s, 2H, C_6H_2), 7.74 (s, 1H, CH=C). ^{13}C NMR (126 MHz, DMSO-d_6) δ 26.4 (CH_2), 28.1 (CH_2), 55.0 (OCH_3), 55.3 ($2OCH_3$), 91.0 (2C, C_6H_2), 105.6 (2C, C_6H_2) 106.0 (β-Py[C]–CN), 112.2 (CN), 113.2 (CN), 120.6 (γ-Py[C]–CN), 123.1 (CH=C), 137.5 (CH=C), 141.8 (β-Py[C]–CH_2), 151.0 (α-Py[C]–Cl), 158.8 (MeO[2C]C_6H_2), 162.2 (MeO[C]C_6H_2), 165.8 (α-Py[C]–C). MS (EI): m/z = 381 [M^+ (^{35}Cl)] (77), 383 [M^+ (^{37}Cl)] (27). Anal. Calcd for $C_{20}H_{16}ClN_3O_3$: C, 62.92; H, 4.22; N, 11.01. Found, C, 62.87; H, 4.20; N, 11.07.

(*E*)-2-Chloro-7-((9-ethyl-9*H*-carbazol-3-yl)methylene)-6,7-dihydro-5*H*-cyclopenta[*b*]pyridine-3,4-dicarbonitrile (**1g**). Red crystals. Yield 76%. M.p. 316-317 °C (dec.). ^{1}H NMR (500 MHz, DMSO-d_6) δ 1.32–1.39 (m, 3H, CH_3), 2.75–3.04–3.34 (m, >4H, $2CH_2$, HDO), 4.40–4.51 (m, 2H, NCH_2), 7.20–7.28 (m, 1H, carbazol), 7.46–7.53 (m, 1H, carbazol), 7.57–7.80 (m, 5H, carbazole, CH=C), 8.11–8.23 (m, 1H, carbazol), 8.30–8.46 (m, 1H, carbazol). ^{13}C NMR (126 MHz, DMSO-d_6) δ 13.4 (CH_3), 27.3 (CH_2), 28.2 (CH_2), 37.4 (NCH_2), 106.6 (β-Py[C]–CN), 109.4, 109.5, 112.7 (CN), 113.6 (CN), 119.5, 120.5 (γ-Py[C]–CN), 122.5, 122.6, 123.1 (CH=C), 126.3, 126.8, 128.3, 131.6, 134.7, 137.0 (CH=C), 140.3, 140.4, 141.9 (β-Py[C]–CH_2), 151.6 (α-Py[C]–Cl), 165.8 (α-Py[C]–C). MS (EI): m/z = 408 [M^+ (^{35}Cl)] (100), 410 [M^+ (^{37}Cl)] (36). Anal. Calcd for $C_{25}H_{17}ClN_4$: C, 73.44; H, 4.19; N, 13.70. Found, C, 73.56; H, 4.22; N, 13.64.

(*E*)-2-Chloro-7-(4-(diphenylamino)benzylidene)-6,7-dihydro-5*H*-cyclopenta[*b*]pyridine-3,4-dicarbonitrile (**1h**). Black crystals. Yield 90%. M.p. 261-263 °C (dec.).^{1}H NMR (500 MHz, DMSO-d_6) δ 3.17–3.22 (m, 2H, CH_2), 3.23–3.28 (m, 2H, CH_2), 6.94 (d, J = 8.7 Hz, 2H, C_6H_4), 7.12 (d, J = 7.6 Hz, 4H, C_6H_5), 7.16 (t, J = 7.4 Hz, 2H, C_6H_5), 7.38 (t, J = 7.8 Hz, 4H, C_6H_5), 7.52 (s, 1H, CH=C), 7.57 (d, J = 8.7 Hz, 2H, C_6H_4). ^{1}H NMR (500 MHz, $CDCl_3$) δ 3.22–3.30 (m, 4H, CH_2), 7.05 (d, J = 8.7 Hz, 2H, C_6H_4), 7.12 (d, J = 7.4 Hz, 4H, C_6H_5), 7.16 (t, J = 7.7 Hz, 2H, C_6H_5), 7.32 (t, J = 7.8 Hz, 4H, C_6H_5), 7.42 (d, J = 8.7 Hz, 2H, C_6H_4), 7.66 (s, 1H, CH=C). ^{13}C NMR (126 MHz, DMSO-d_6) δ 27.1 (CH_2), 28.0 (CH_2), 105.9 (β-Py[C]–CN), 112.9 (CN), 113.9 (CN), 120.5 (2C, C_6H_4), 121.0 (γ-Py[C]–CN), 124.4 (2C, C_6H_5), 125.3 (4C, C_6H_5), 128.4 (1C, C_6H_4), 129.4 (CH=C), 129.8 (4C, C_6H_5), 131.6 (2C, C_6H_4), 135.4 (CH=C), 142.1 (β-Py[C]–CH_2), 146.1 (2C, N[C]C_6H_5), 148.3 (1C, N[C]C_6H_4), 151.4 (α-Py[C]–Cl), 166.1 (α-Py[C]–C). MS (EI): m/z = 458 [M^+ (^{35}Cl)] (100), 460 [M^+ (^{37}Cl)] (38). Anal. Calcd for $C_{29}H_{19}ClN_4$: C, 75.89; H, 4.17; N, 12.21. Found, C, 76.02; H, 4.14; N, 12.17.

(*E*)-2-Chloro-7-(4-(dimethylamino)benzylidene)-6,7-dihydro-5*H*-cyclopenta[*b*]pyridine-3,4-dicarbonitrile (**1i**). Violet crystals. Yield 93%. M.p. 295-297 °C (dec.).^{1}H NMR (500 MHz, DMSO-d_6) δ 3.02 (s, 6H, $(CH_3)_2N$), 3.13–3.26 (m, >4H, $2CH_2$, HDO), 6.77–6.82 (m, 2H, C_6H_4), 7.49–7.54 (m, 3H, C_6H_4, CH=C). ^{13}C NMR (126 MHz, DMSO-d_6) δ 26.7 (CH_2), 27.6 (CH_2), 39.0 (2C, $(CH_3)_2N$), 103.9 (β-Py[C]–CN), 111.6 (2C, C_6H_4), 112.3 (CN), 113.3 (CN), 122.9 (γ-Py[C]–CN), 130.7 (CH=C), 131.4 (2C, C_6H_4), 131.8 (1C, C_6H_4), 135.3 (CH=C), 141.4 (β-Py[C]–CH_2), 149.8 (1C, N[C]C_6H_4), 150.6 (α-Py[C]–Cl), 167.1 (α-Py[C]–C). MS (EI): m/z = 334 [M^+ (^{35}Cl)] (100), 336 [M^+ (^{37}Cl)] (34). Anal. Calcd for $C_{19}H_{15}ClN_4$: C, 68.16; H, 4.52; N, 16.73. Found, C, 68.09; H, 4.49; N, 16.77.

2. Solvatochromic studies data

Table S1. Solvatochromic properties of compound **1c**

Solvent	λ_{abs}, nm[a]	ε, M^{-1} cm^{-1}	λ_{em}, nm[b]	Stokes shift		Φ_{em},%[c]
				nm	cm^{-1}	
CCl_4	448	28000	475	27	1269	85.9
toluene	443	22900	500	57	2573	87.5
1,4-dioxane	432	28400	509	77	3502	79.9
AcOEt	443	27700	538	95	3986	60.1
AcOH	432	26200	530	98	4280	72.9
CH_2Cl_2	431	29700	559	128	5313	47.5
DMSO	439	26400	578	139	5478	48.4
MeCN	434	33600	558	124	5120	20.4
HCOOH	438	30500	588	150	5824	2.2
MeOH	431	–[d]	565	134	5503	26.5

[a]Absorption maxima were registered in solution (10^{-5} M)
[b]Emission maxima were registered in solution (10^{-5} M) (absorption maxima were used for excitation)
[c]Relative emission quantum yield (Φ_{em}) was estimated using solution of fluorescein in 0.01 M KOH in ethanol (Φ_{em} 97% at λ_{ex} 425 nm) as standard.
[d]Poorly soluble sample

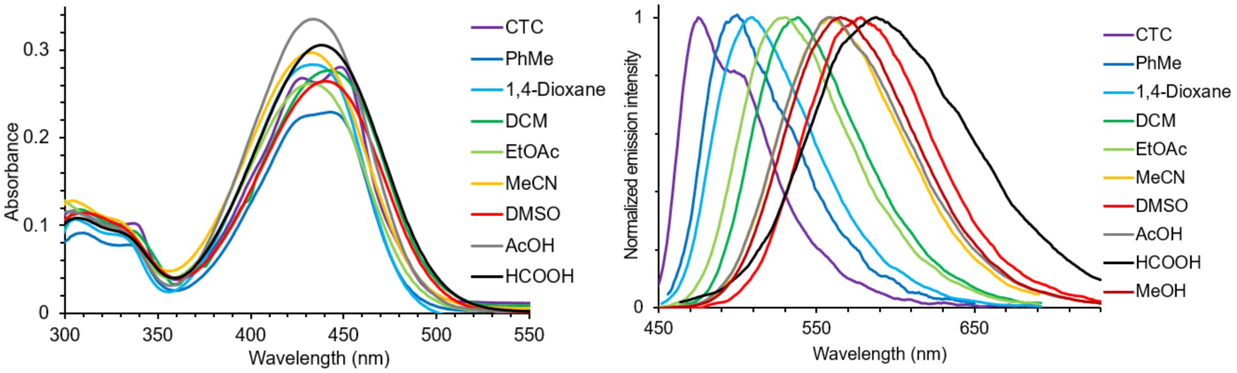

Figure S1. Absorption (left) and normalized emission spectra (right) of compound **1c** in various solvents (10^{-5} M).

3. Titration experiment data

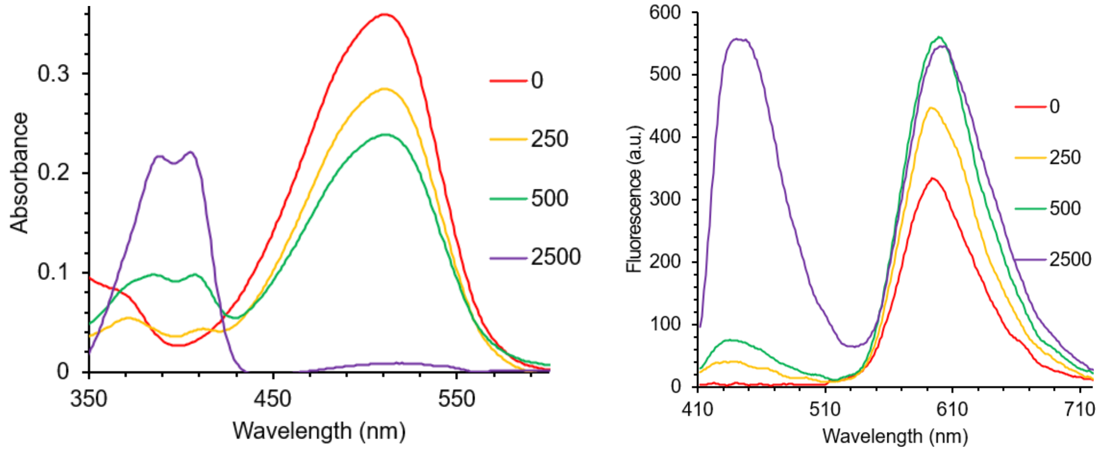

A **B**

Figure S2. Absorption (A) and normalized emission spectra (B) of compound **1i** in toluene (10^{-5} M) upon the addition of given equivalents of trifluoroacetic acid.

4. Lippert–Mataga plots

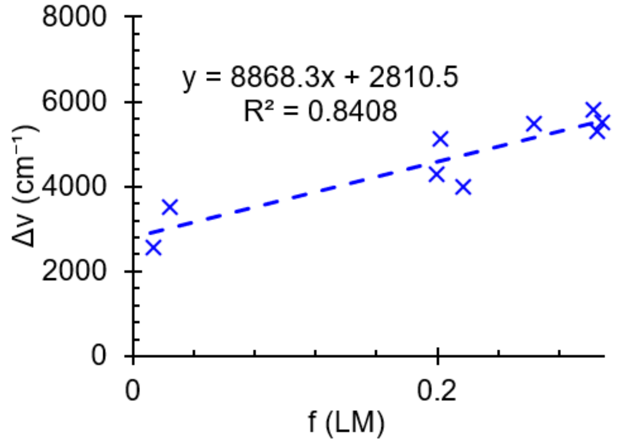

Figure S3. The Lippert–Mataga plot for compound **1c**.

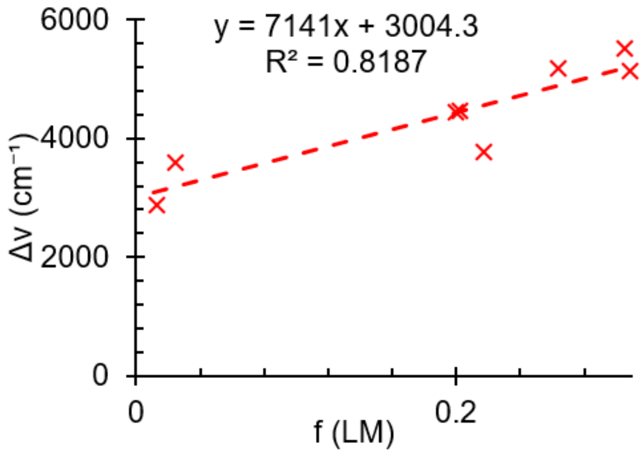

Figure S4. The Lippert–Mataga plot for compound **1i**.

5. Kawski–Chamma–Viallet's equation

Table S2. Solvent parameters, Kawski–Chamma–Viallet (KCV) solvent polarity function and absorption/emission maxima wavenumbers of compounds **1c** and **1i**.

Solvent	κ	n_D^{20}	f (KCV)	Compound 1c			Compound 1i		
				ν_a (cm^{-1})	ν_e (cm^{-1})	$\frac{(\nu_a + \nu_e)}{2}$ (cm^{-1})	ν_a (cm^{-1})	ν_e (cm^{-1})	$\frac{(\nu_a + \nu_e)}{2}$ (cm^{-1})
CCl_4	2.24	1.4601	0.392	22321	21053	21687	19417	18051	18734
toluene	2.38	1.4969	0.437	22573	20000	21287	19608	16722	18165
1,4-dioxane	2.25	1.4224	0.362	23148	19646	21397	19881	16287	18084
AcOEt	6.02	1.3724	0.522	23148	18868	21008	19802	15361	17581
AcOH	6.15	1.3716	0.525	23041	17921	20481	19569	15106	17338
CH_2Cl_2	8.93	1.4241	0.634	22573	18587	20580	19011	15244	17128
DMSO	46.7	1.4783	0.822	22779	17301	20040	19048	13870	16459
MeCN	37.5	1.3441	0.676	23202	17889	20545	19608	14085	16846
MeOH	32.7	1.3284	0.653	23202	17699	20450	19608	14472	17040

Equation S1. The Kawski–Chamma–Viallet (KCV) solvent polarity function.

$$f\ (KCV) = \frac{2n^2+1}{2(n^2+2)} \times \left(\frac{\kappa-1}{\kappa+2} - \frac{n^2-1}{n^2+2}\right) + \frac{3(n^4-1)}{2(n^2+2)^2}$$

where: κ is the relative permittivity and *n* is the refraction index of the solvents

Equation S2. The Kawski–Chamma–Viallet (KCV) equation.

$$\frac{(\nu_a+\nu_e)}{2} = f\ (KCV)\frac{2({\mu_E}^2 - {\mu_G}^2)}{hca^3} + const$$

where: μ are dipole moments in Debyes denoted with subscripts '*E*' and '*G*' for excited and ground state respectively

$h = 6.6262\times10^{-27}$ erg·s (Planck's constant)

$c = 2.9979\times10^{10}$ cm/s (the speed of light in vacuum)

a = 6.3 Å (the radius of the cavity taken the half of the distance between electron donor and acceptor fragments)

6. Comparison of photo-physical properties of stilbazoles 1 and A

Table S3. Comparison of photo-physical properties of stilbazoles **1** and **A** in toluene.

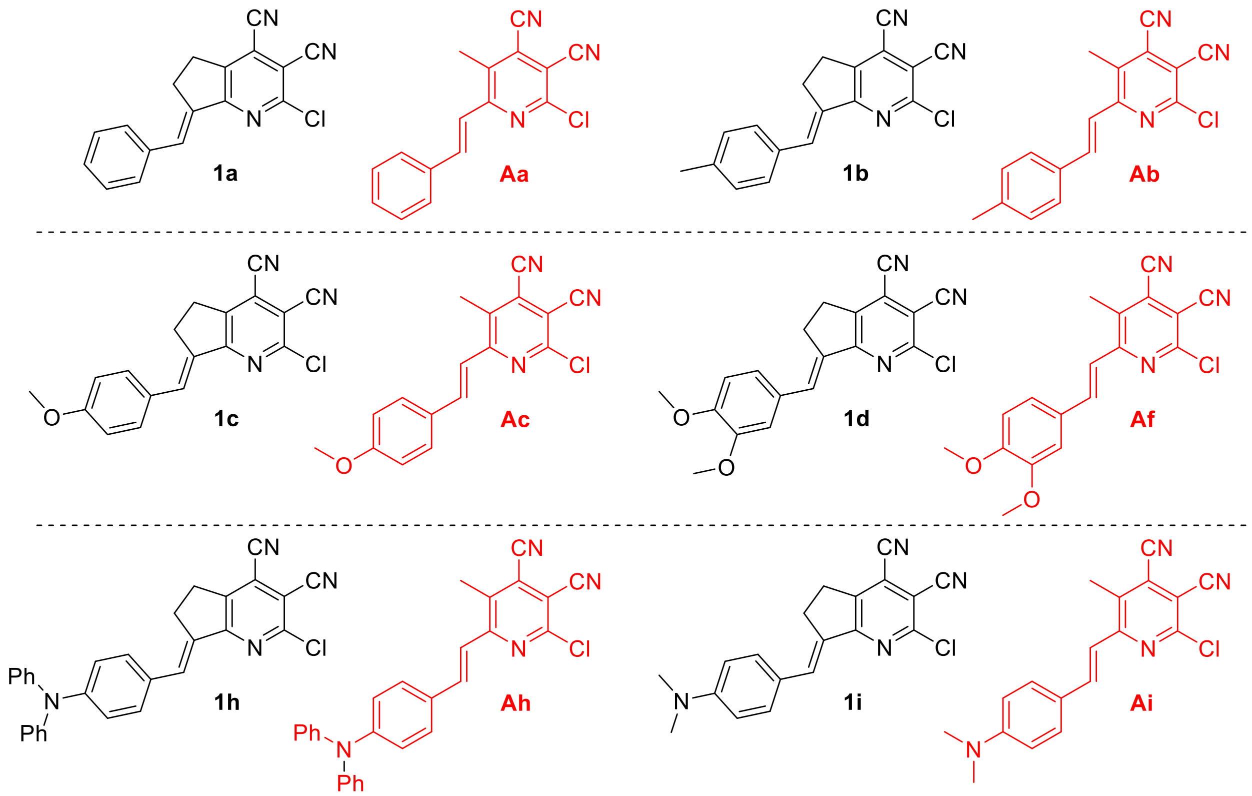

Compound	λ_{abs}, nm[a]	ε, M^{-1} cm^{-1}	λ_{em}, nm[b]	Stokes shift, cm^{-1}	Φ_{em},%[c]
**1a**	402	13100	459	3089	**32.9**
**Aa**	382	30906	442	3554	3.0
**1b**	411	23500	470	3054	**12.2**
**Ab**	390	18224	453	3566	3.2
**1c**	443	22900	500	2573	**87.5**
**Ac**	409	16404	482	3703	3.9
**1d**	454	13600	520	2796	**35.8**
**Af**	425	12193	508	3844	11.5
**1h**	509	29400	582	2464	**55.2**
**Ah**	493	32224	579	3013	53.9
**Ai**	510	36700	598	2885	**49.5**
**Ai**	495	15032	583	3049	49.3

The data for stilbazoles ***A*** *(highlighted in red) are taken from our previously published work [4].*

Table S4. Comparison of photo-physical properties of stilbazoles **1** and **A** in DMSO.

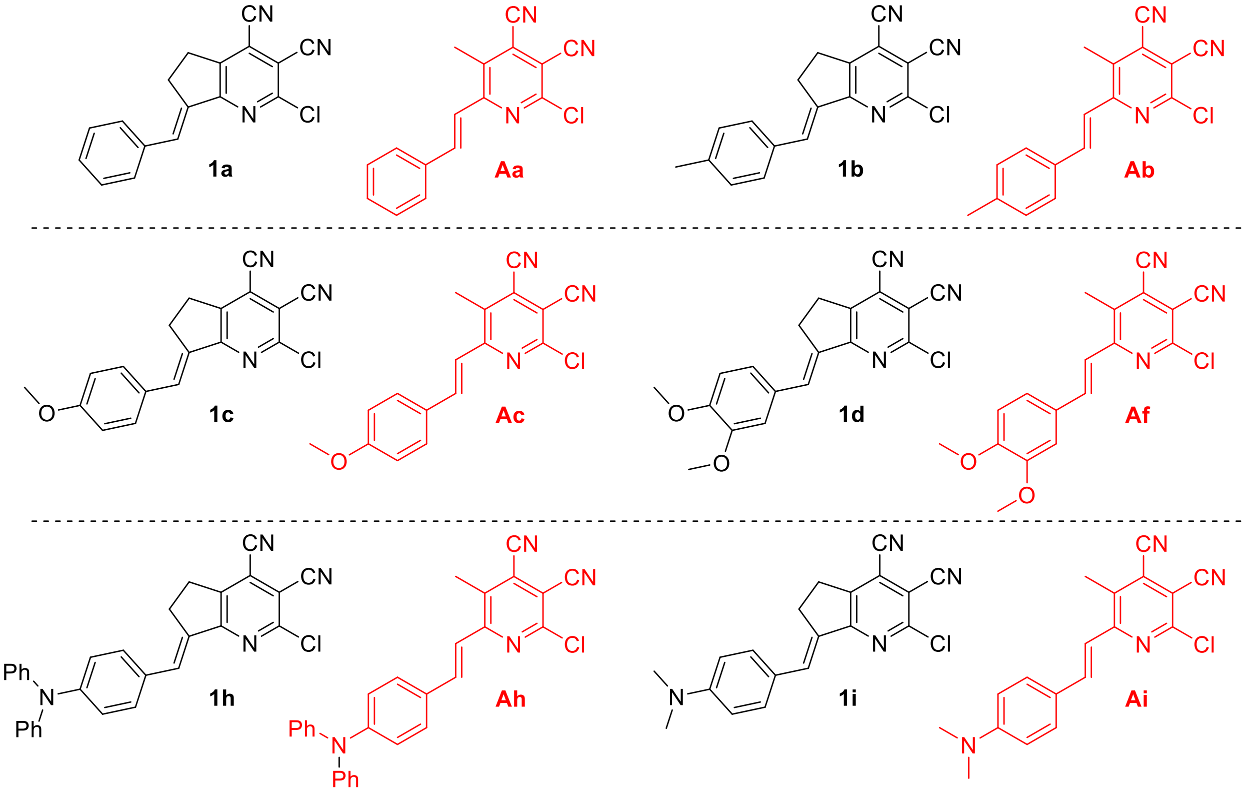

Compound	λ_{abs}, nm	ε, M^{-1} cm^{-1}	λ_{em}, nm	Stokes shift, cm^{-1}	Φ_{em},%
**1a**	409	25600	506	4687	12.3
**Aa**	388	27729	488	5281	1.2
**1b**	419	26300	528	4927	53.4
**Ab**	394	15530	510	5773	4.4
**1c**	439	26400	578	5478	48.4
**Ac**	416	16505	562	6245	14.1
**1d**	460	22100	602	5128	20.5
**Af**	434	16956	611	6675	2.3
**1h**	505	31000	712	5757	0.2
**Ah**	492	31906	655, 697	5058, 5978	0.2
**1i**	525	34900	721	5178	0.6
**Ai**	515	22929	710	5371	0.2

*The data for stilbazoles **A** (highlighted in red) are taken from our previously published work [4].*

7. Copies of ^{1}H and ^{13}C NMR spectra

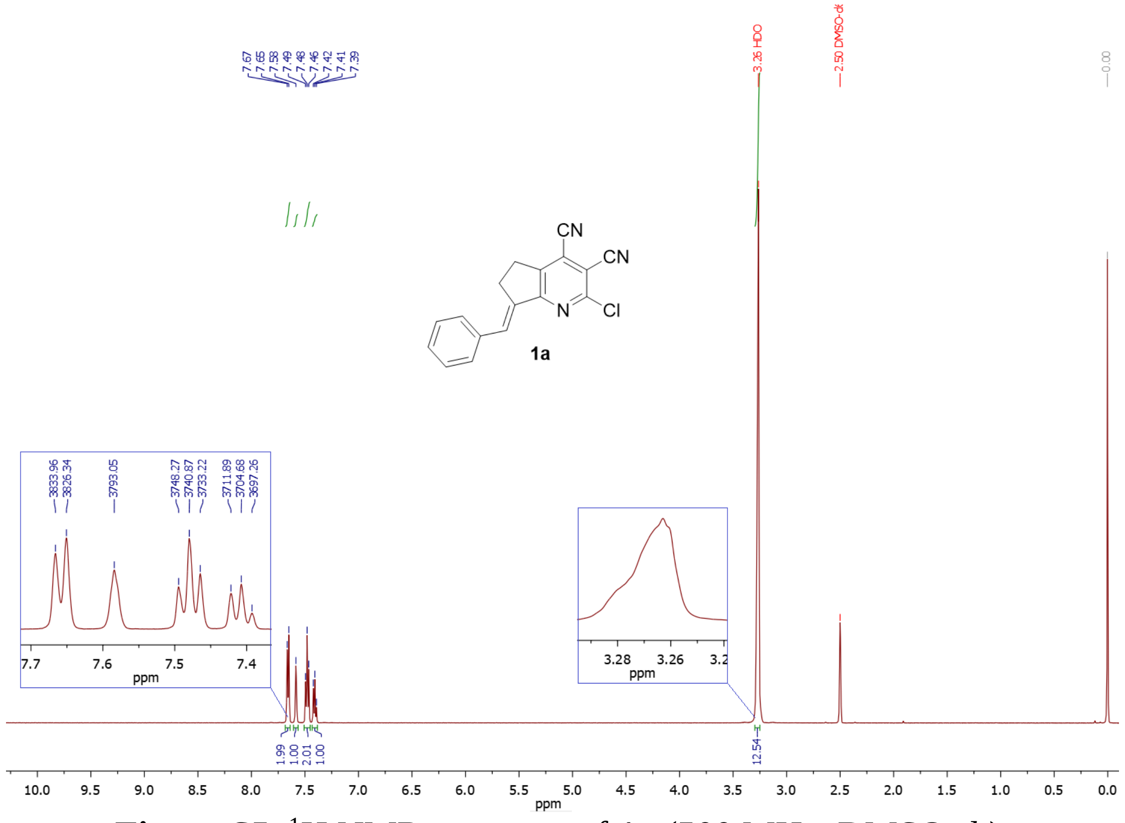

Figure S5. ^{1}H NMR spectrum of **1a** (500 MHz, DMSO-d_6).

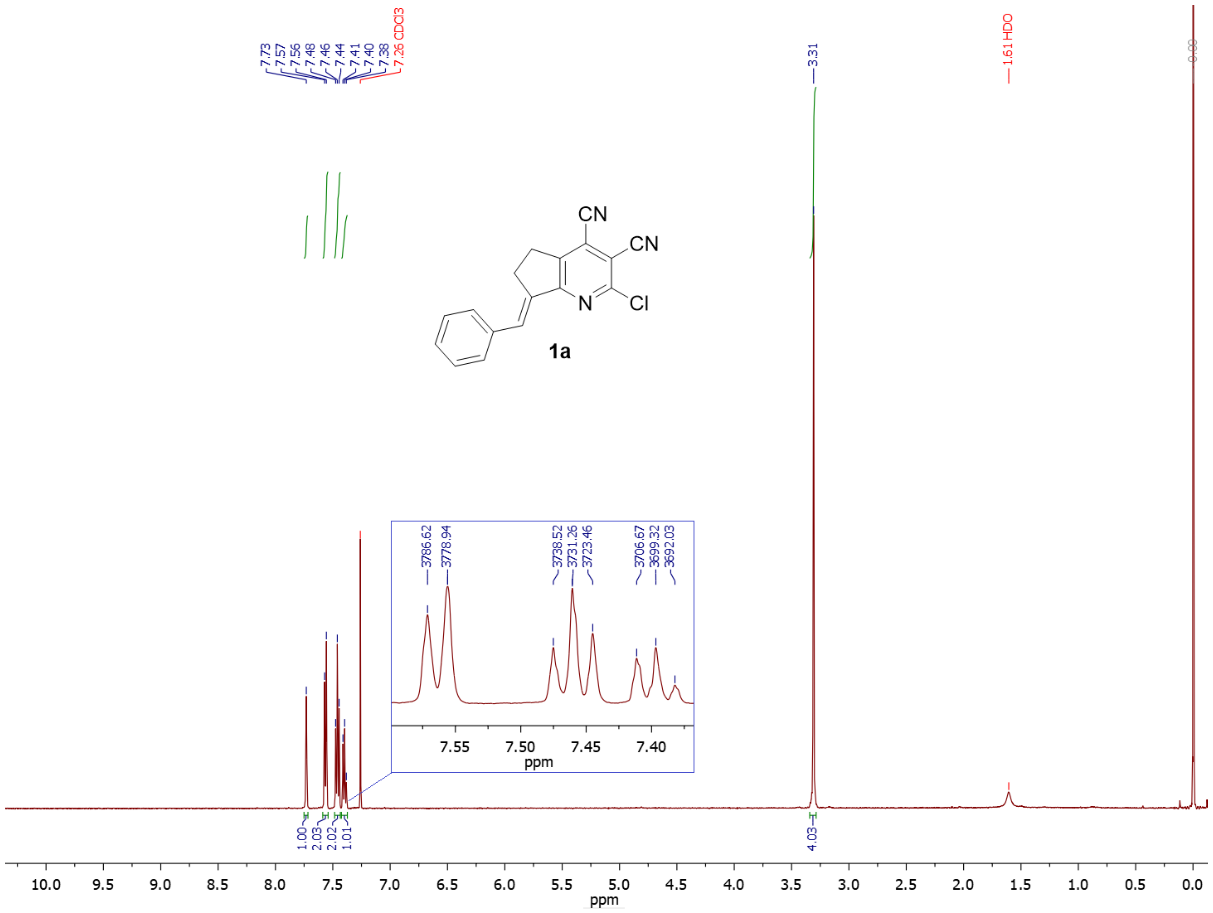

Figure S6. ^{1}H NMR spectrum of **1a** (500 MHz, $CDCl_3$).

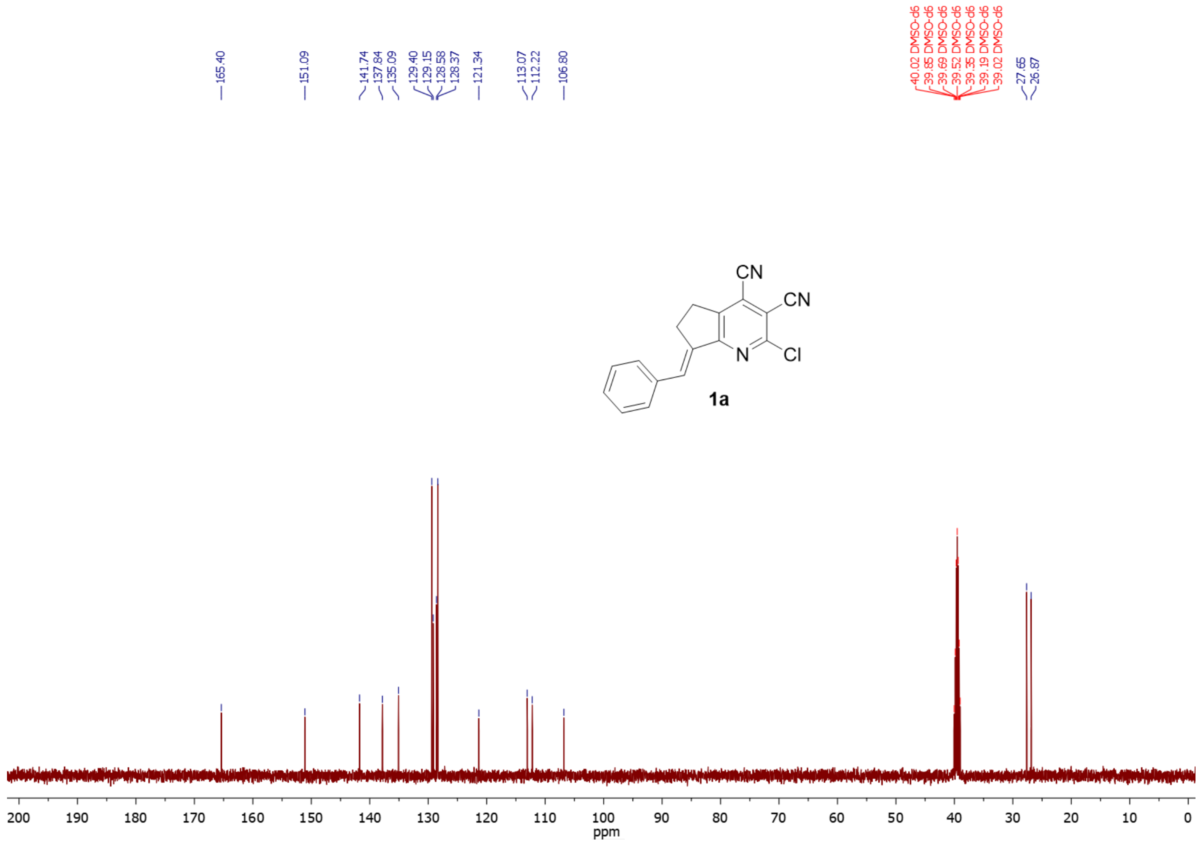

Figure S7. ^{13}C NMR spectrum of **1a** (126 MHz, DMSO-d_6).

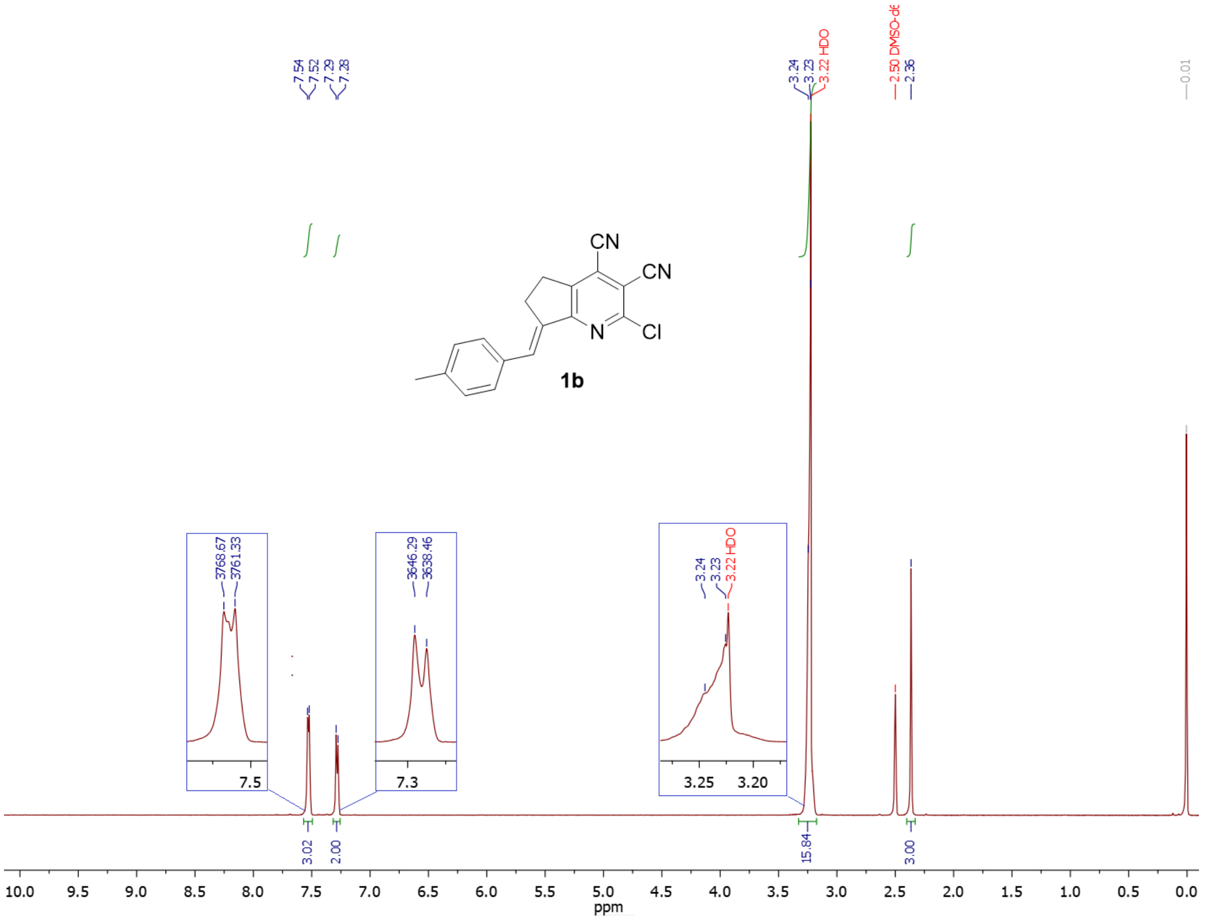

Figure S8. ^{1}H NMR spectrum of **1b** (500 MHz, DMSO-d_6).

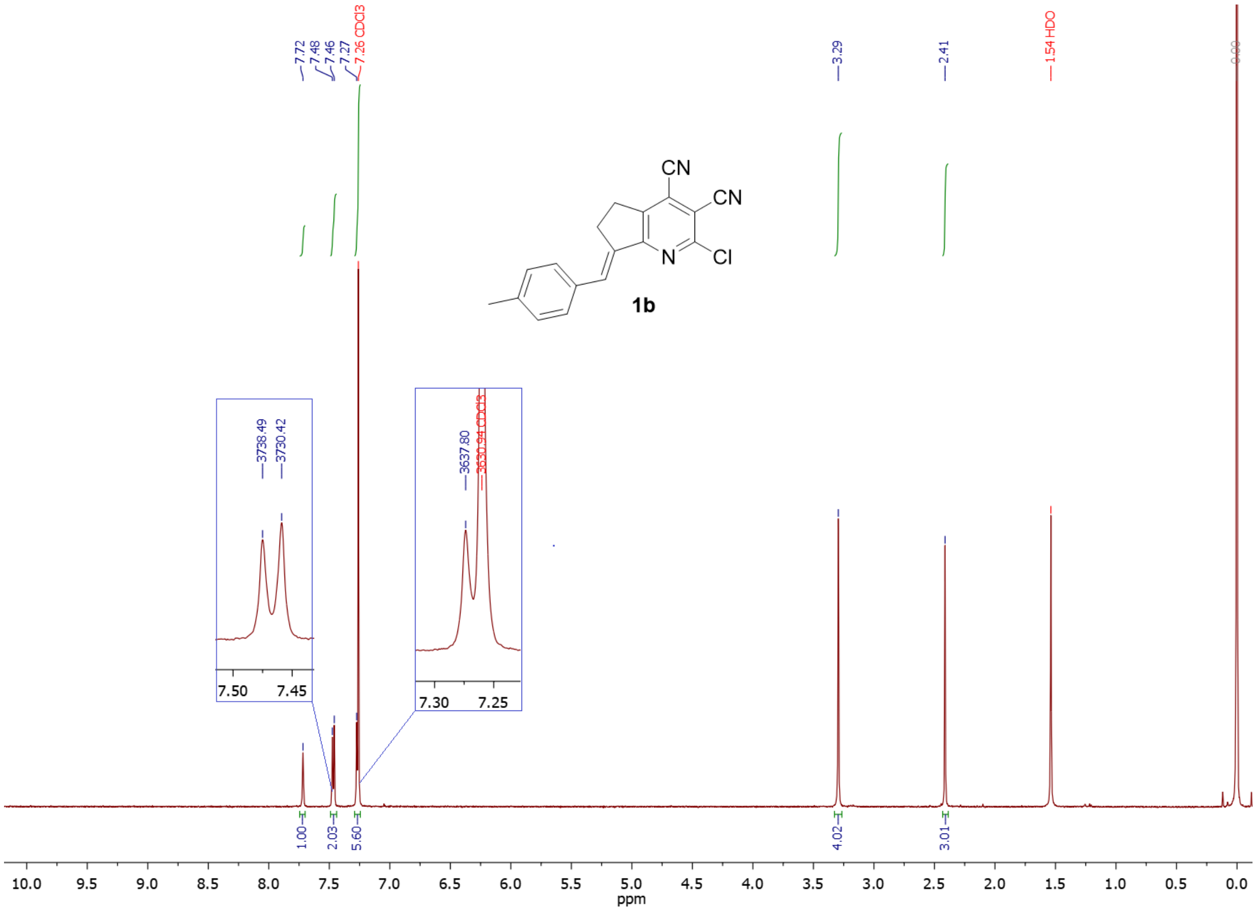

Figure S9. ^{1}H NMR spectrum of **1b** (500 MHz, $CDCl_3$).

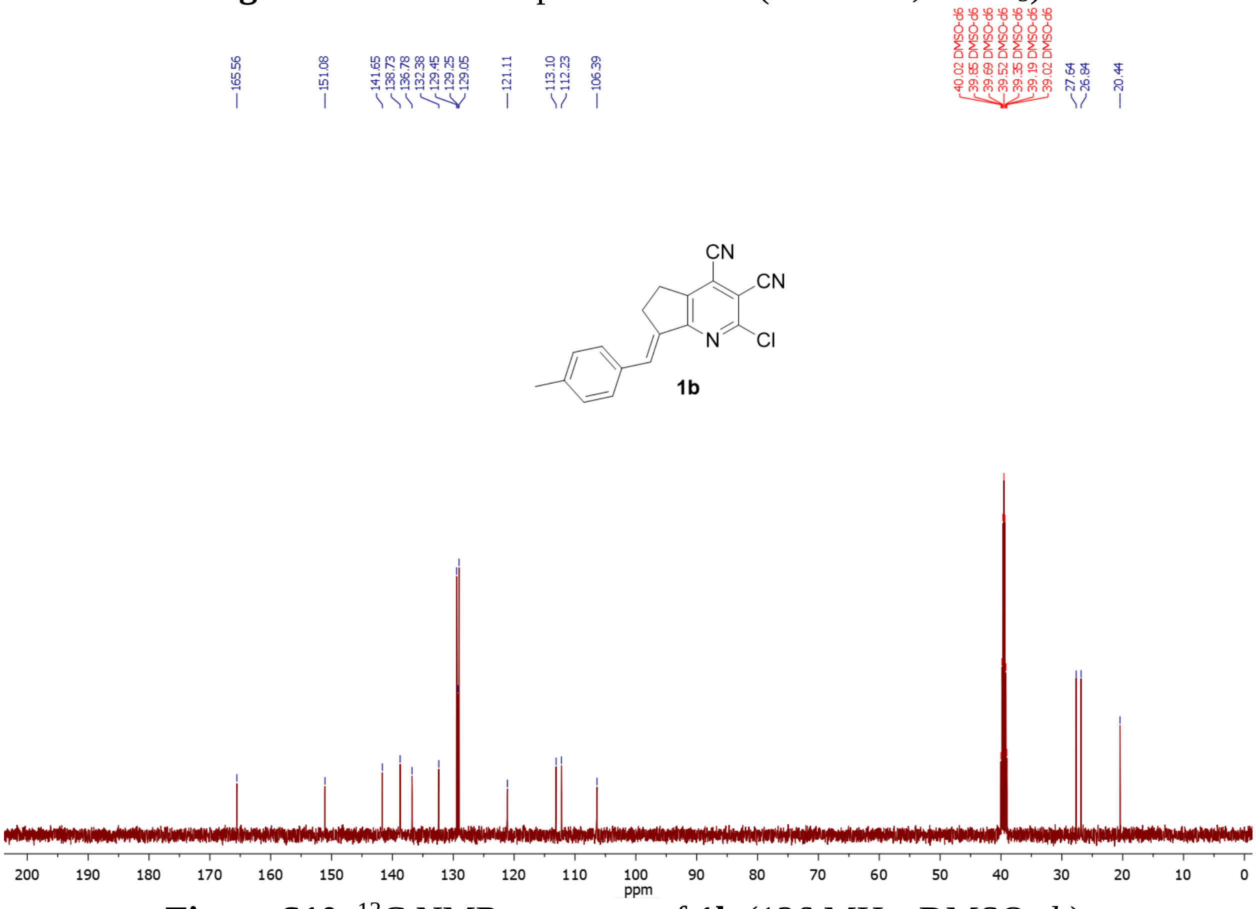

Figure S10. ^{13}C NMR spectrum of **1b** (126 MHz, DMSO-d_6).

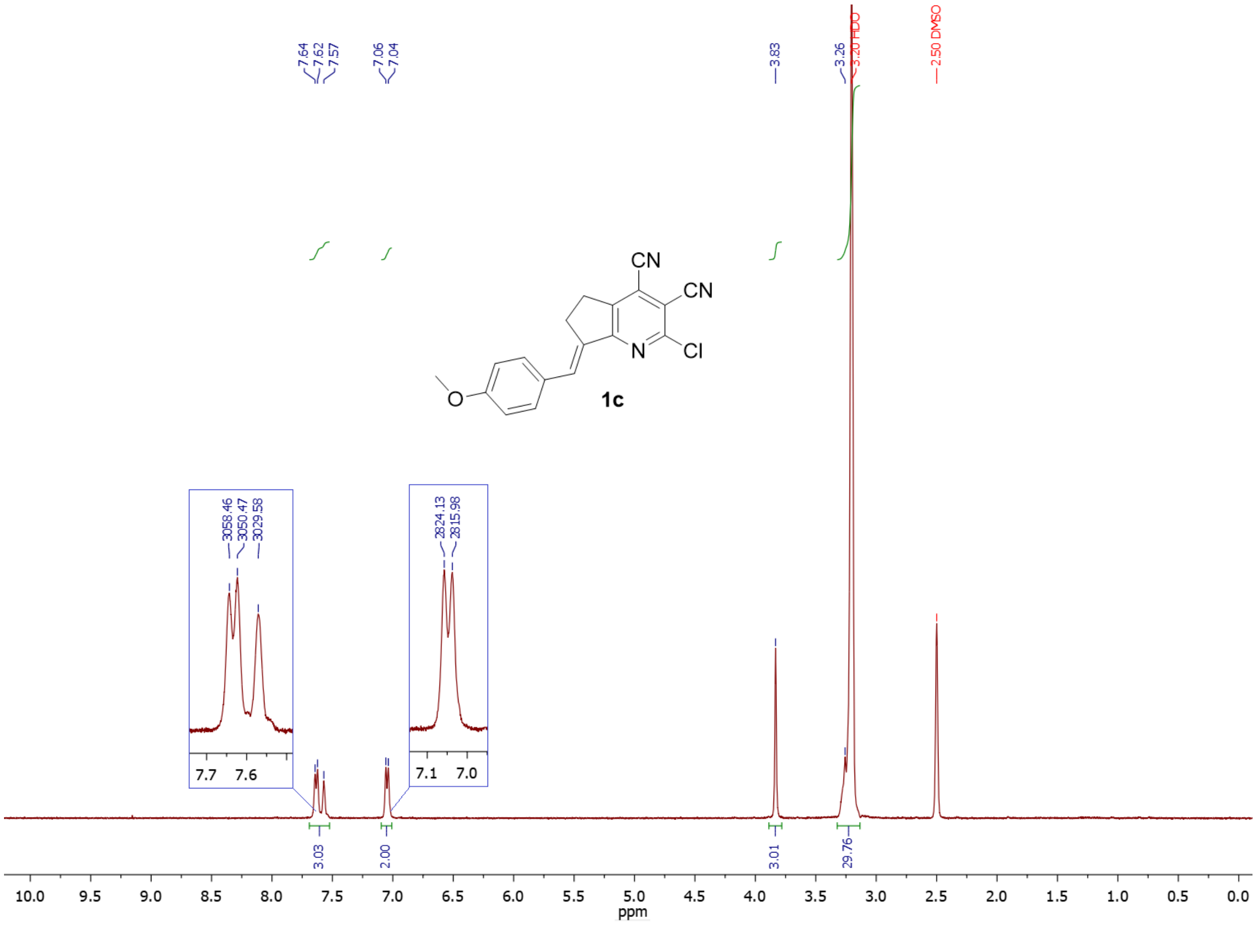

Figure S11. ^{1}H NMR spectrum of **1c** (500 MHz, DMSO-d_6).

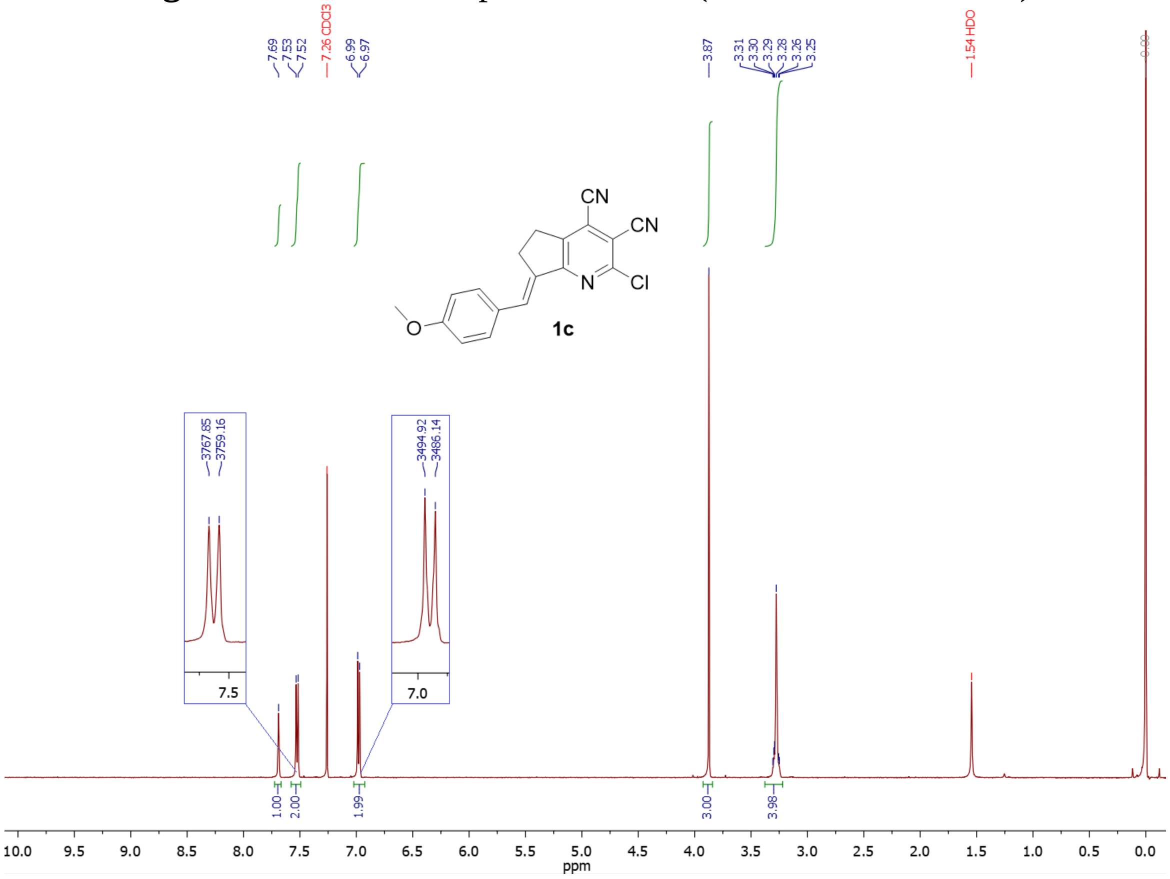

Figure S12. ^{1}H NMR spectrum of **1c** (500 MHz, $CDCl_3$).

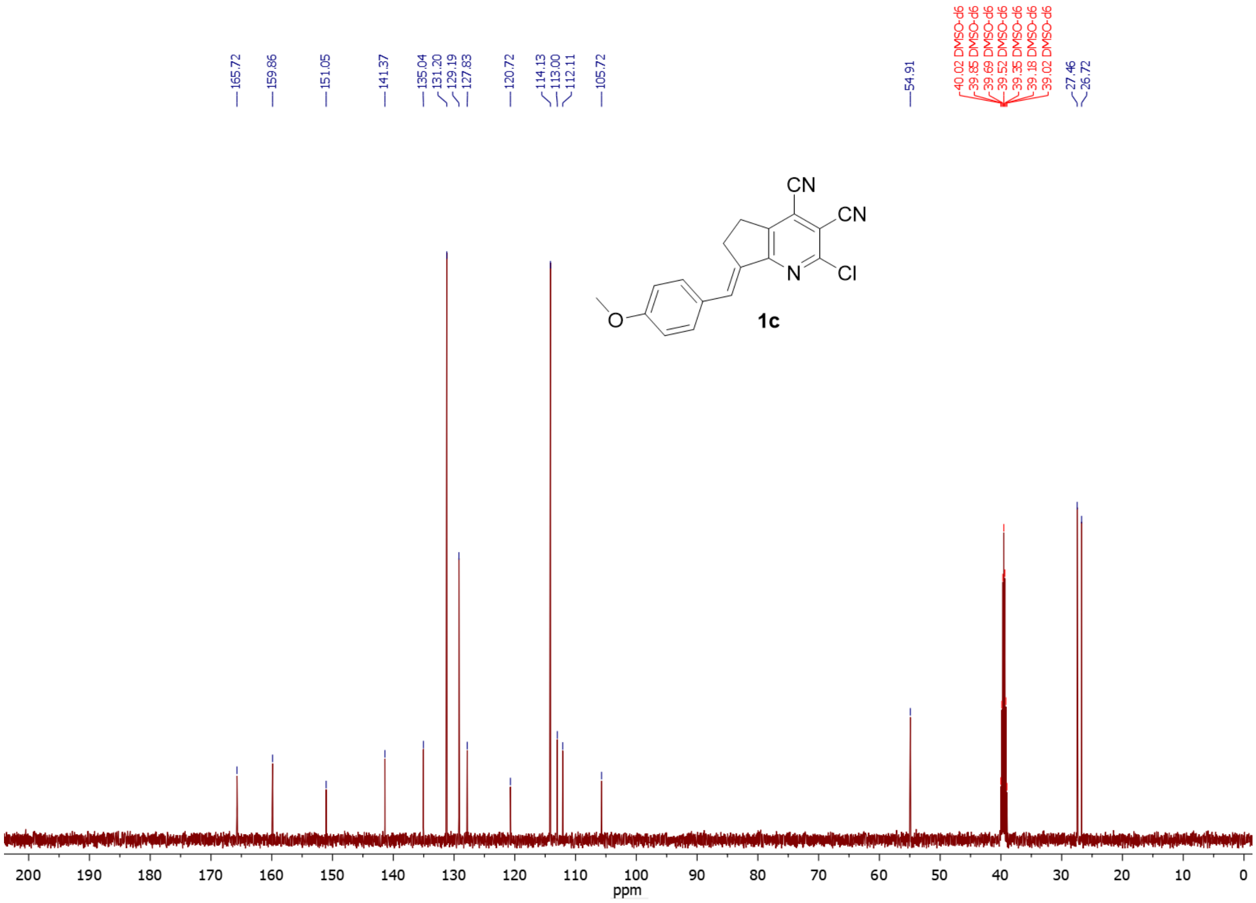

Figure S13. ^{13}C NMR spectrum of **1c** (126 MHz, DMSO-d_6).

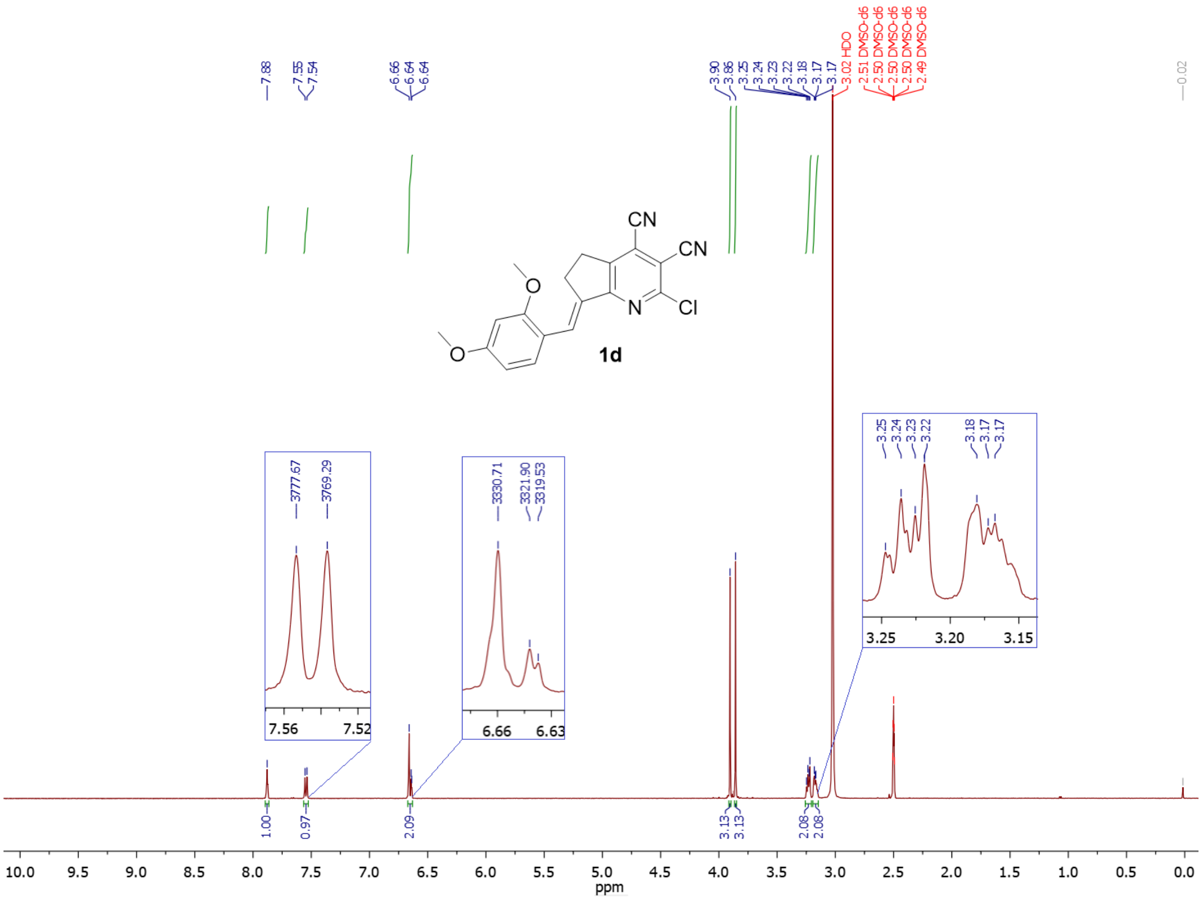

Figure S14. ^{1}H NMR spectrum of **1d** (500 MHz, DMSO-d_6).

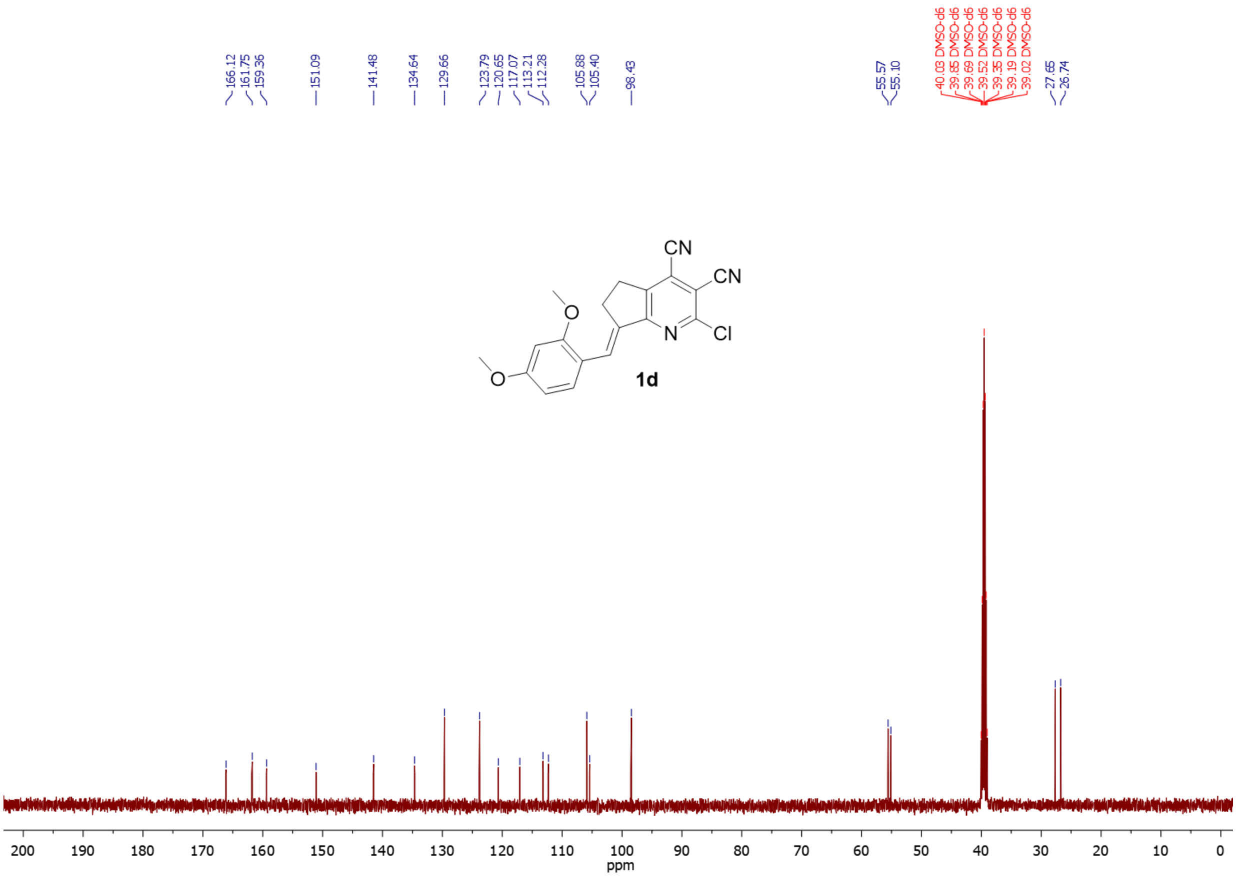

Figure S15. ^{13}C NMR spectrum of **1d** (126 MHz, DMSO-d_6).

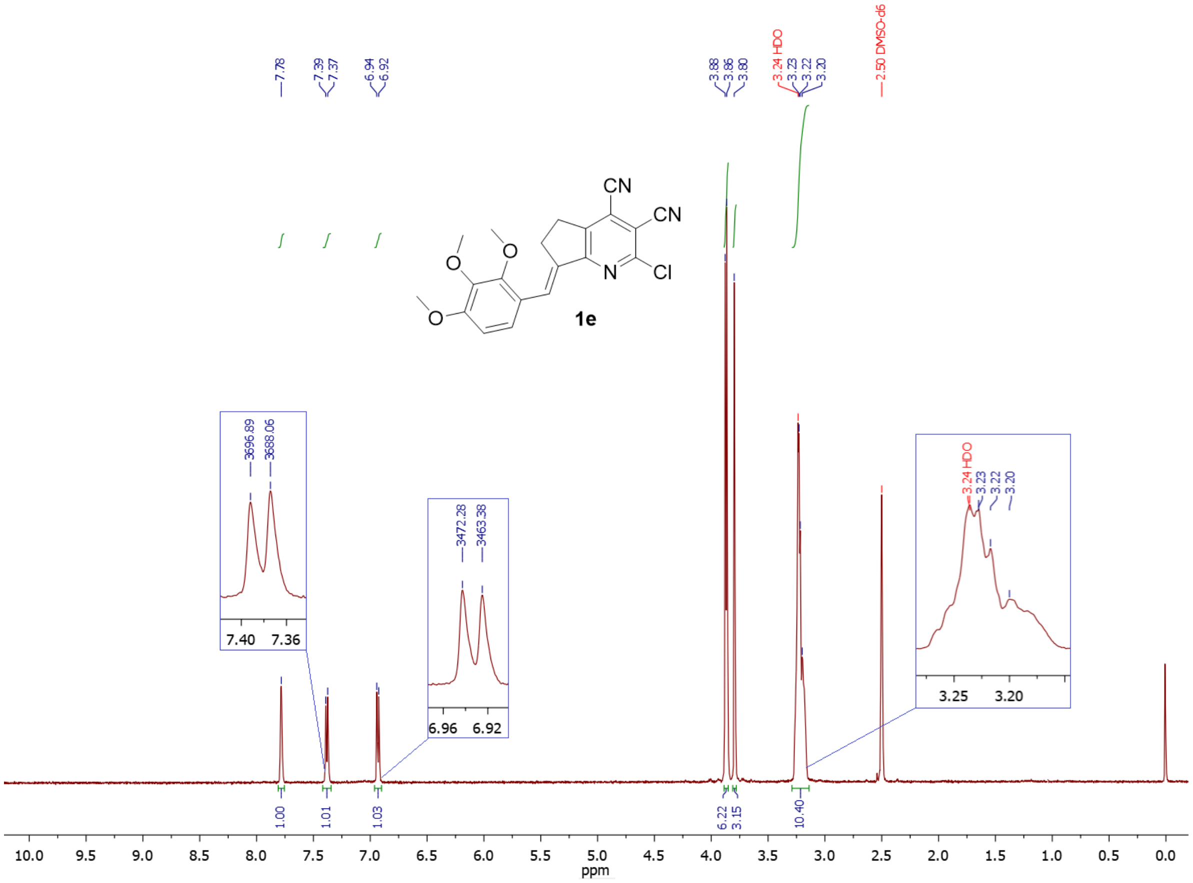

Figure S16. ^{1}H NMR spectrum of **1e** (500 MHz, DMSO-d_6).

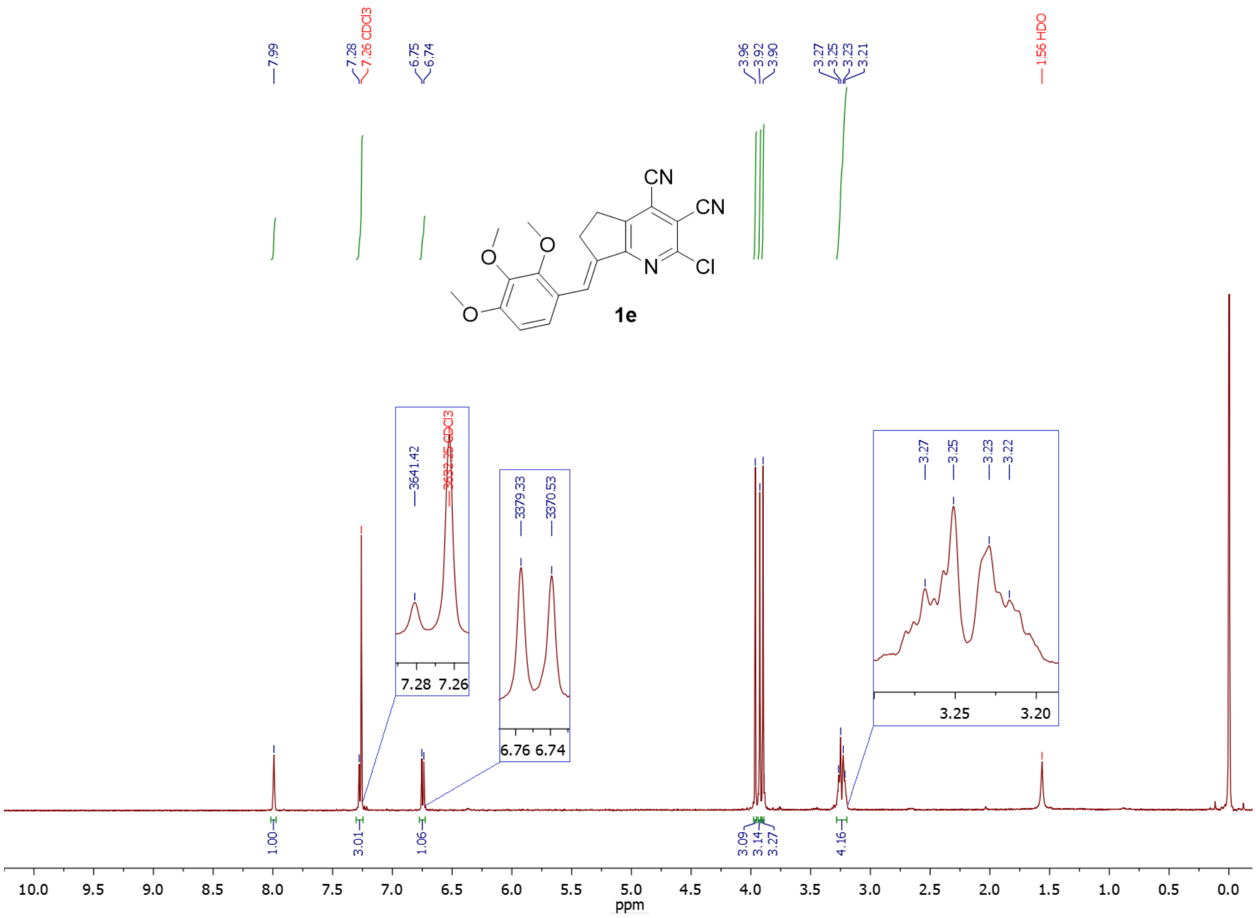

Figure S17. ^{1}H NMR spectrum of **1e** (500 MHz, $CDCl_3$).

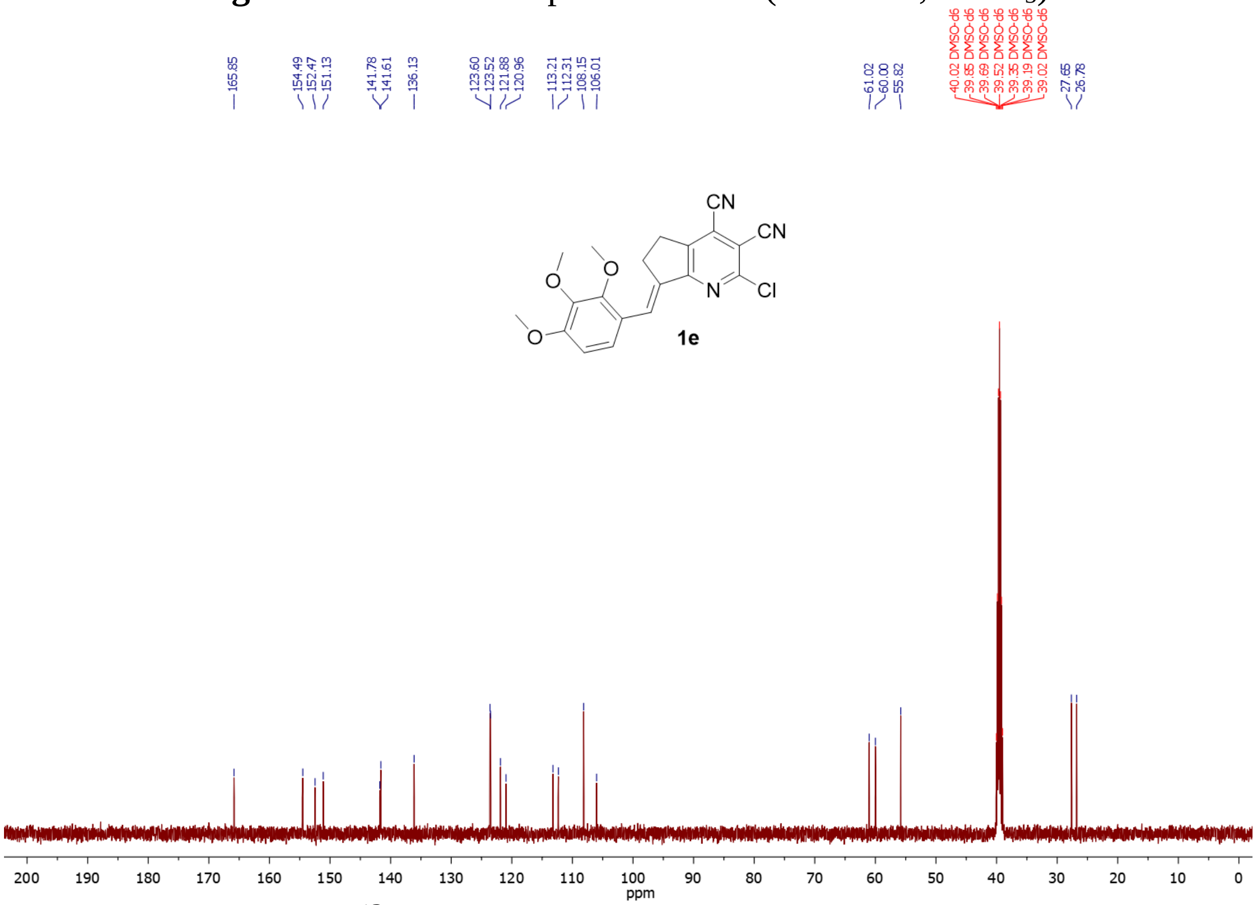

Figure S18. ^{13}C NMR spectrum of **1e** (126 MHz, DMSO-d_6).

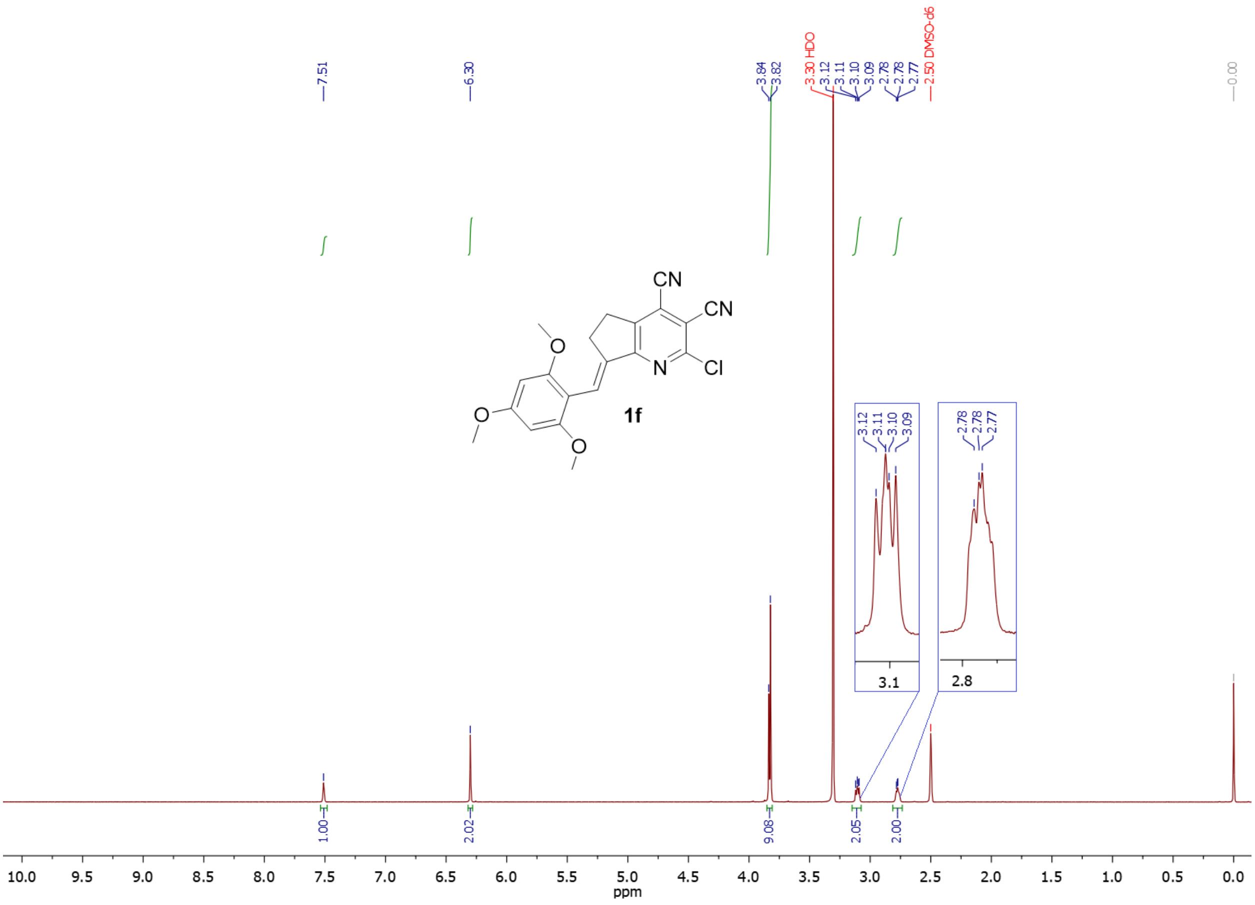

Figure S19. ^{1}H NMR spectrum of **1f** (500 MHz, DMSO-d_6).

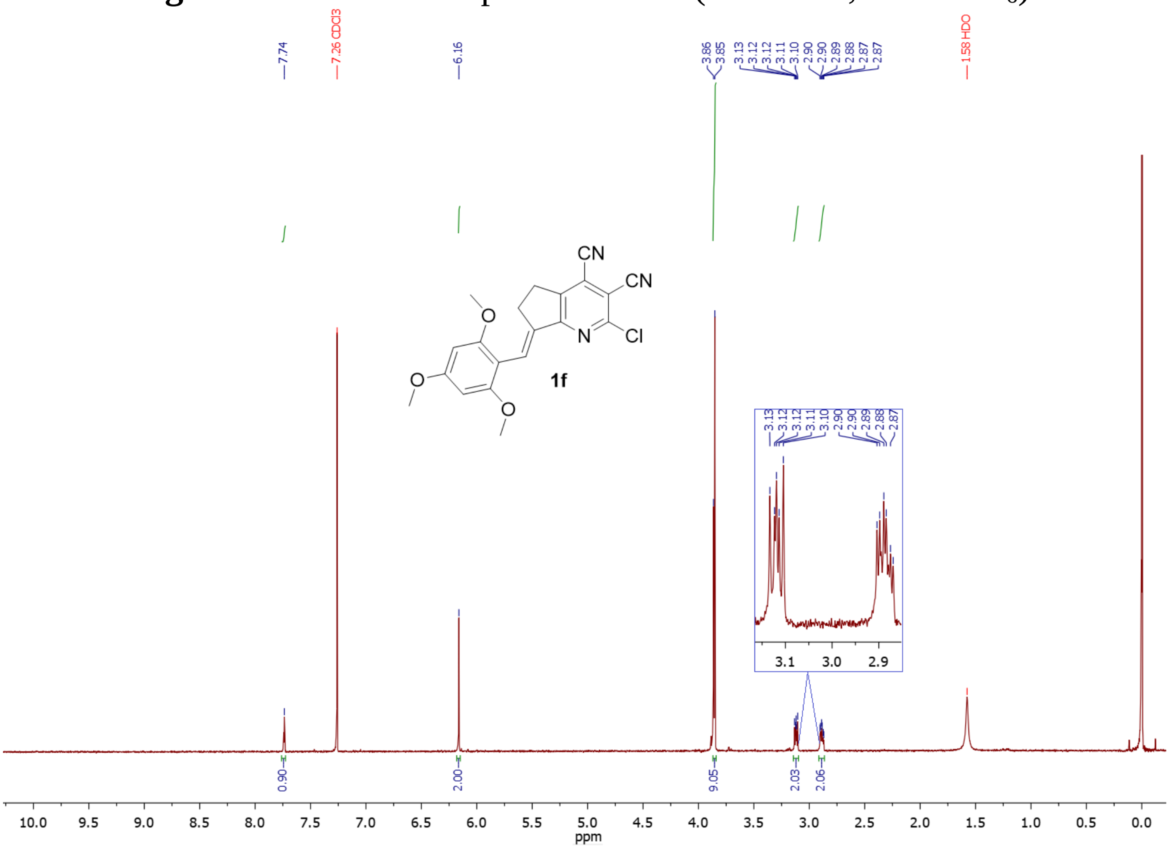

Figure S20. ^{1}H NMR spectrum of **1f** (500 MHz, $CDCl_3$).

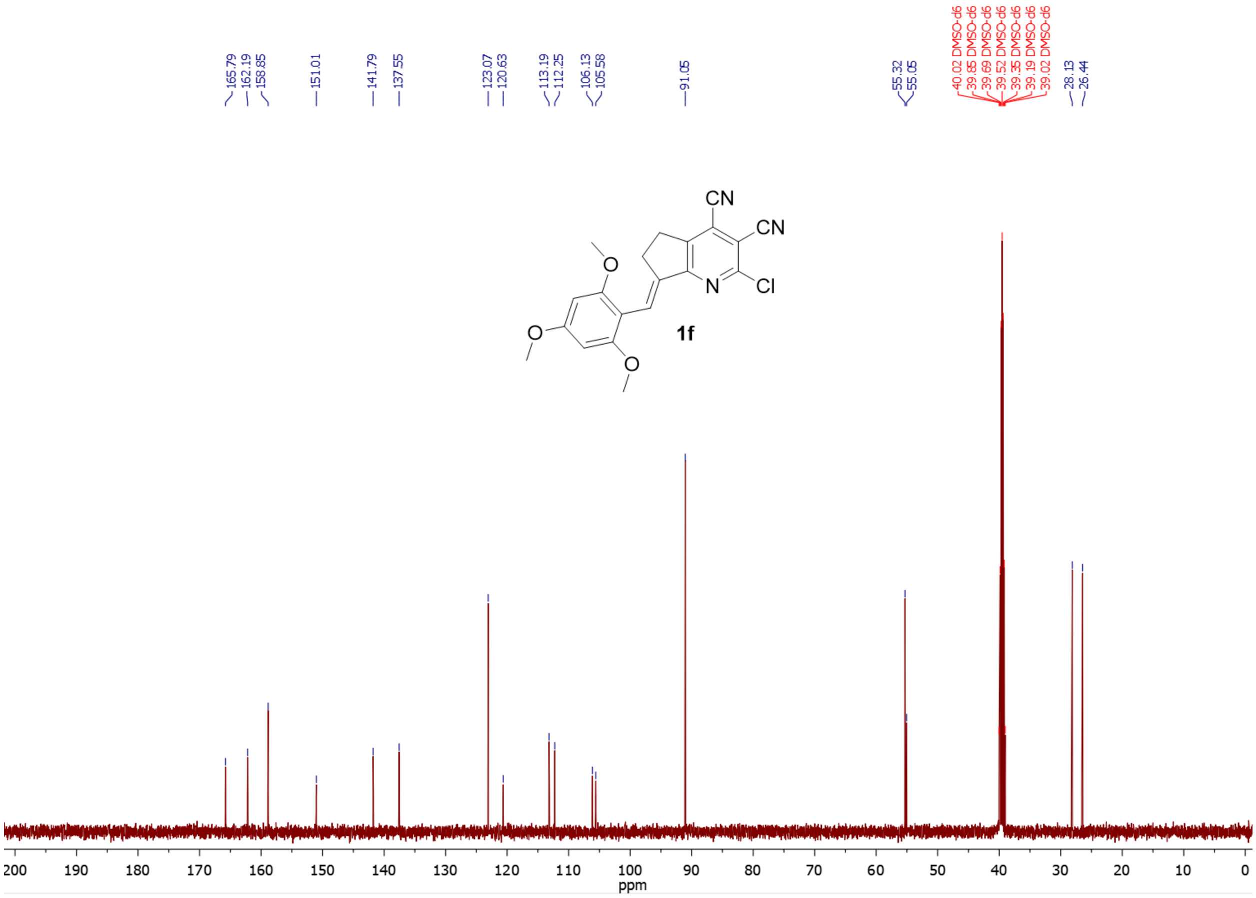

Figure S21. ^{13}C NMR spectrum of **1f** (126 MHz, DMSO-d_6).

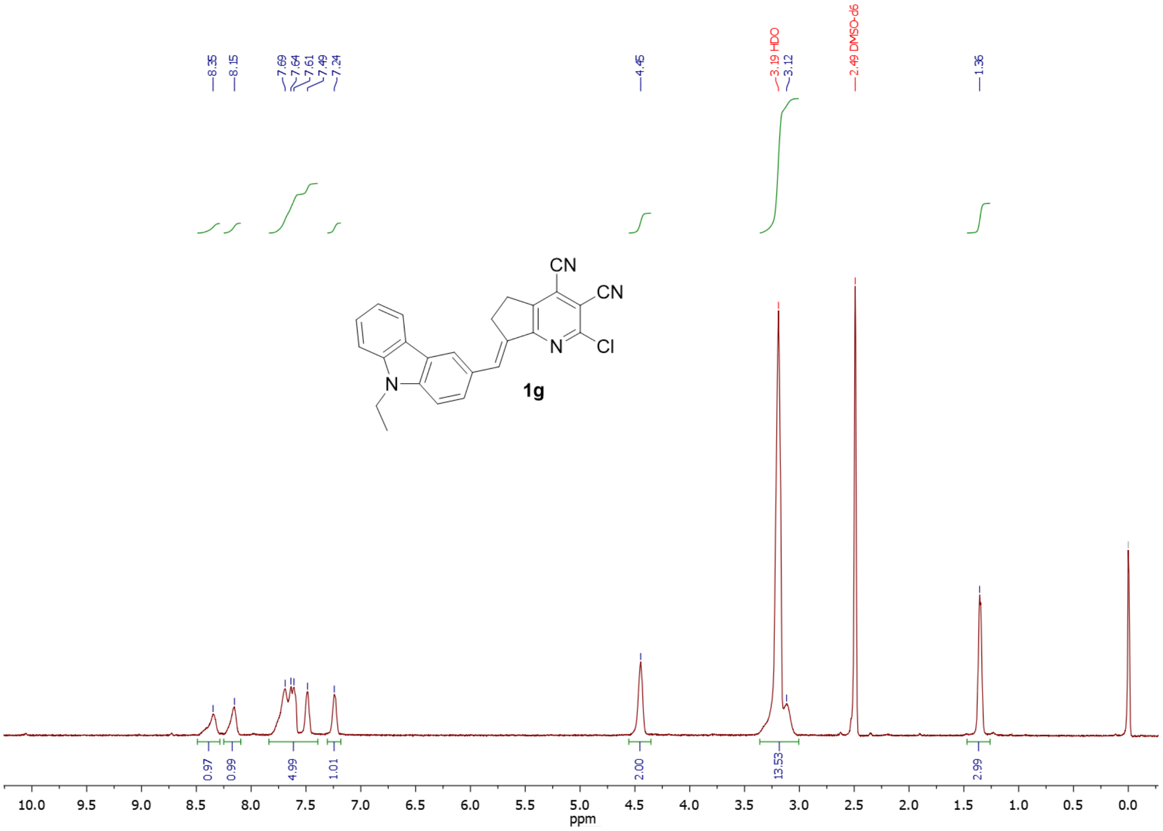

Figure S22. ^{1}H NMR spectrum of **1g** (500 MHz, DMSO-d_6).

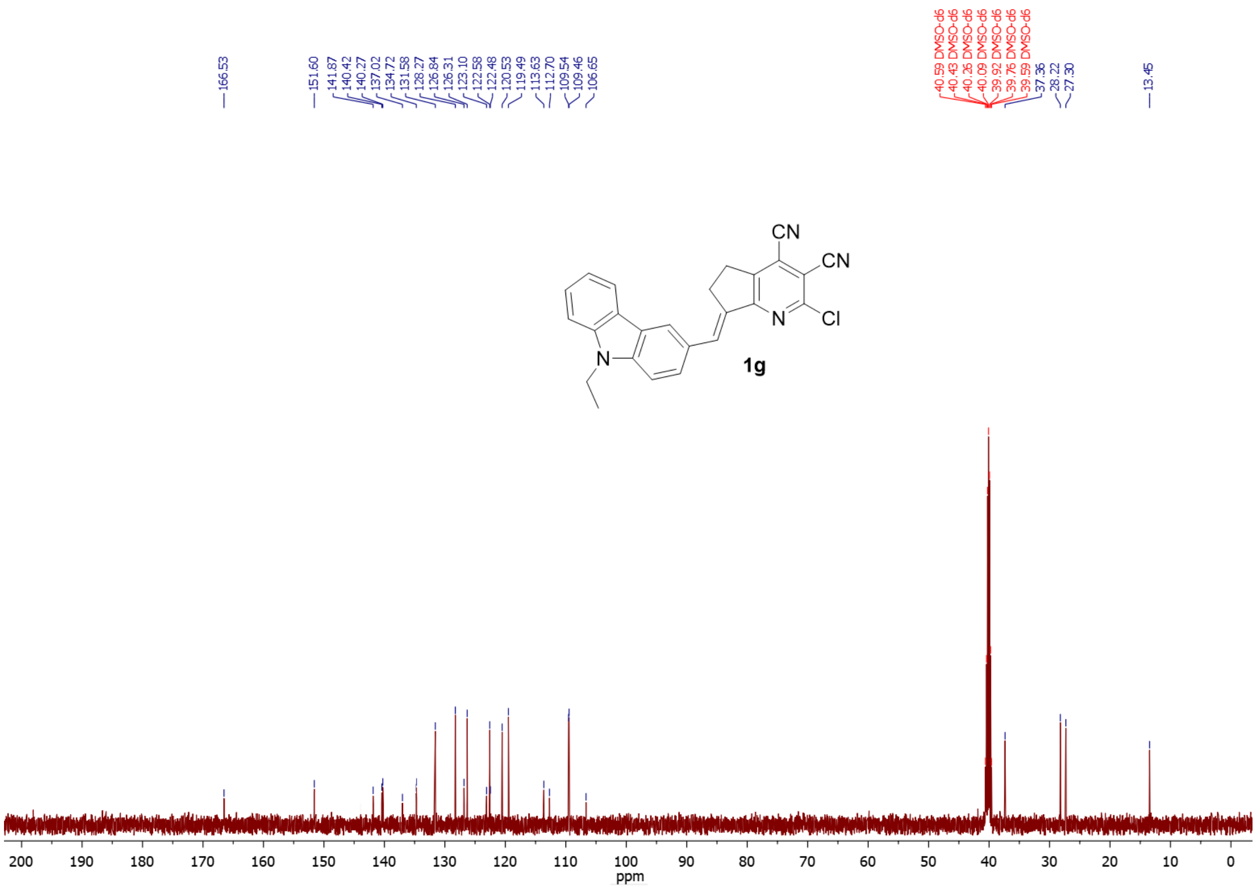

Figure S23. ^{13}C NMR spectrum of **1g** (126 MHz, DMSO-d_6).

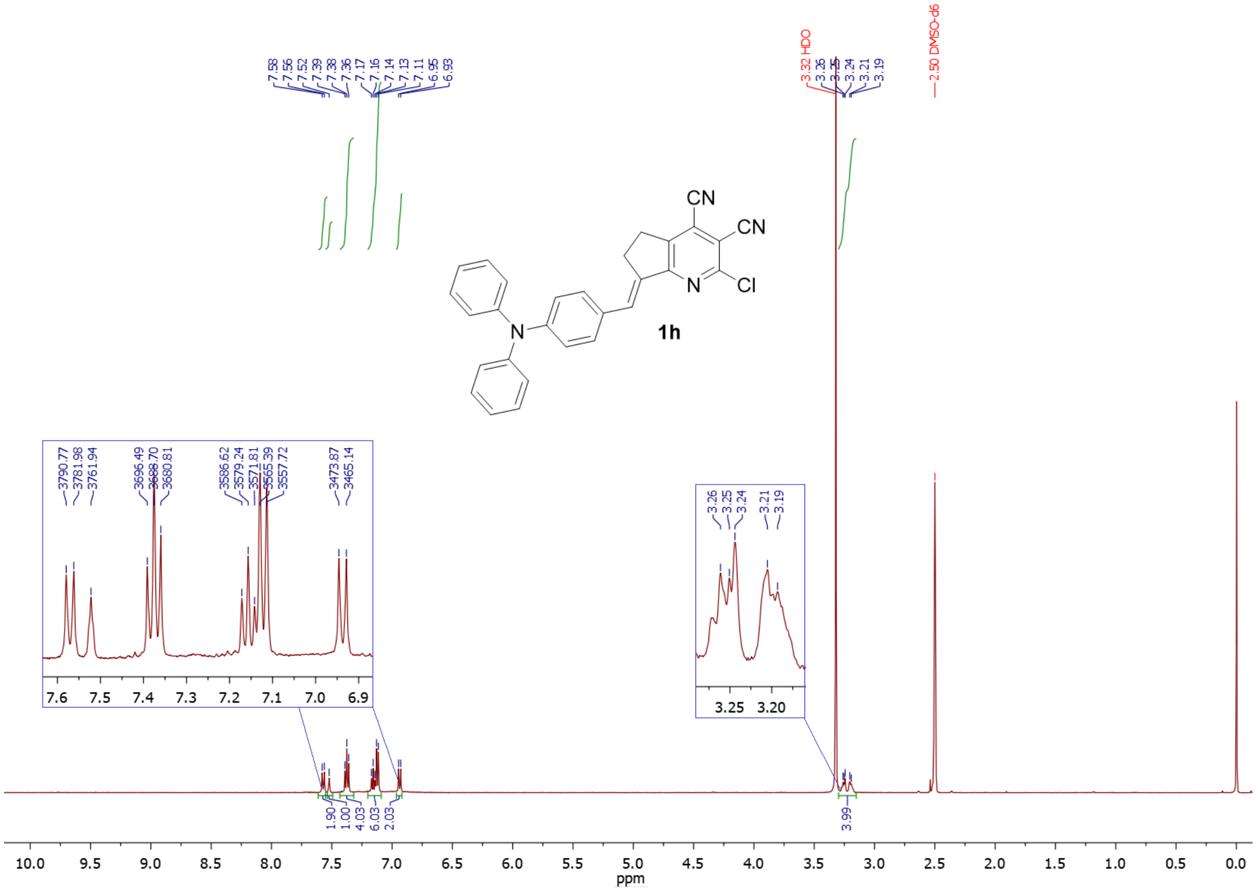

Figure S24. ^{1}H NMR spectrum of **1h** (500 MHz, DMSO-d_6).

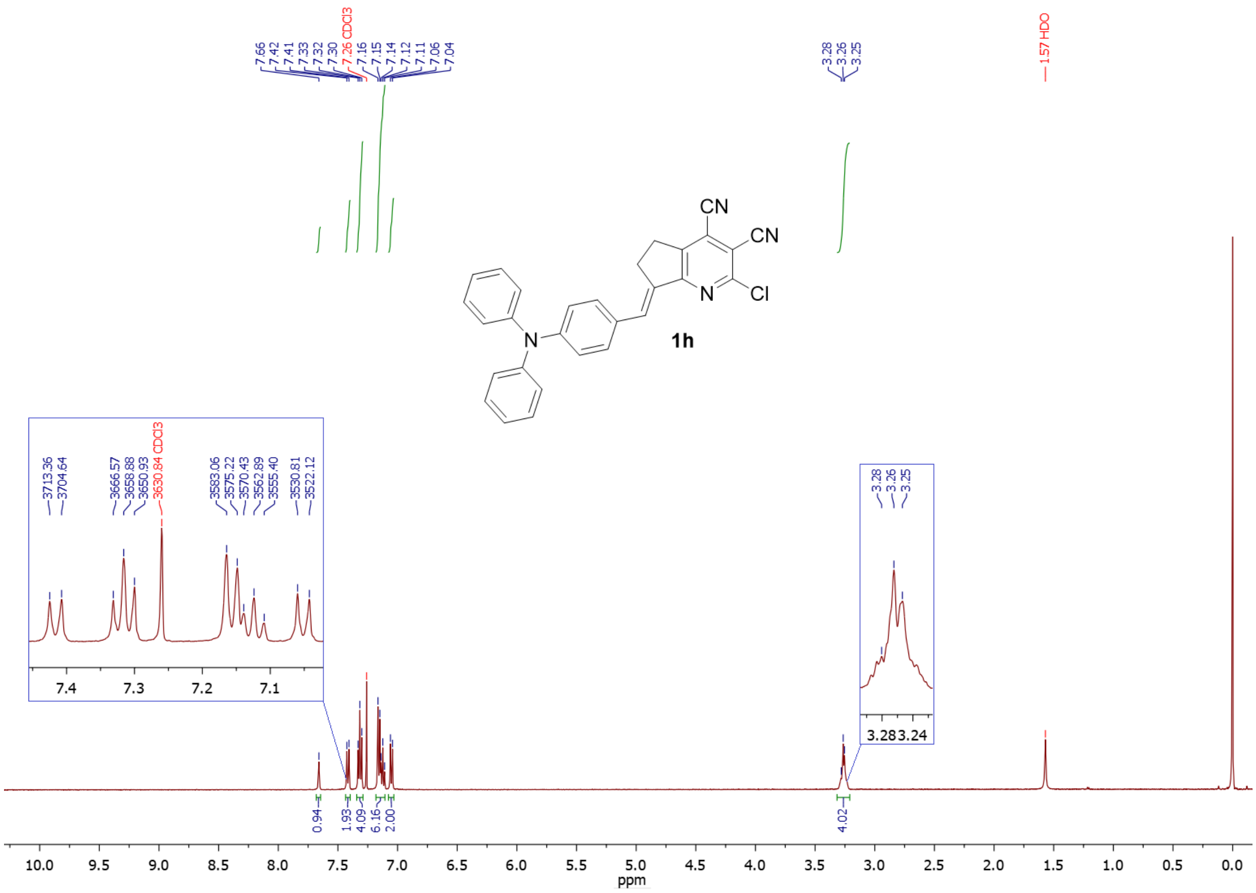

Figure S25. ^{1}H NMR spectrum of **1h** (500 MHz, $CDCl_3$).

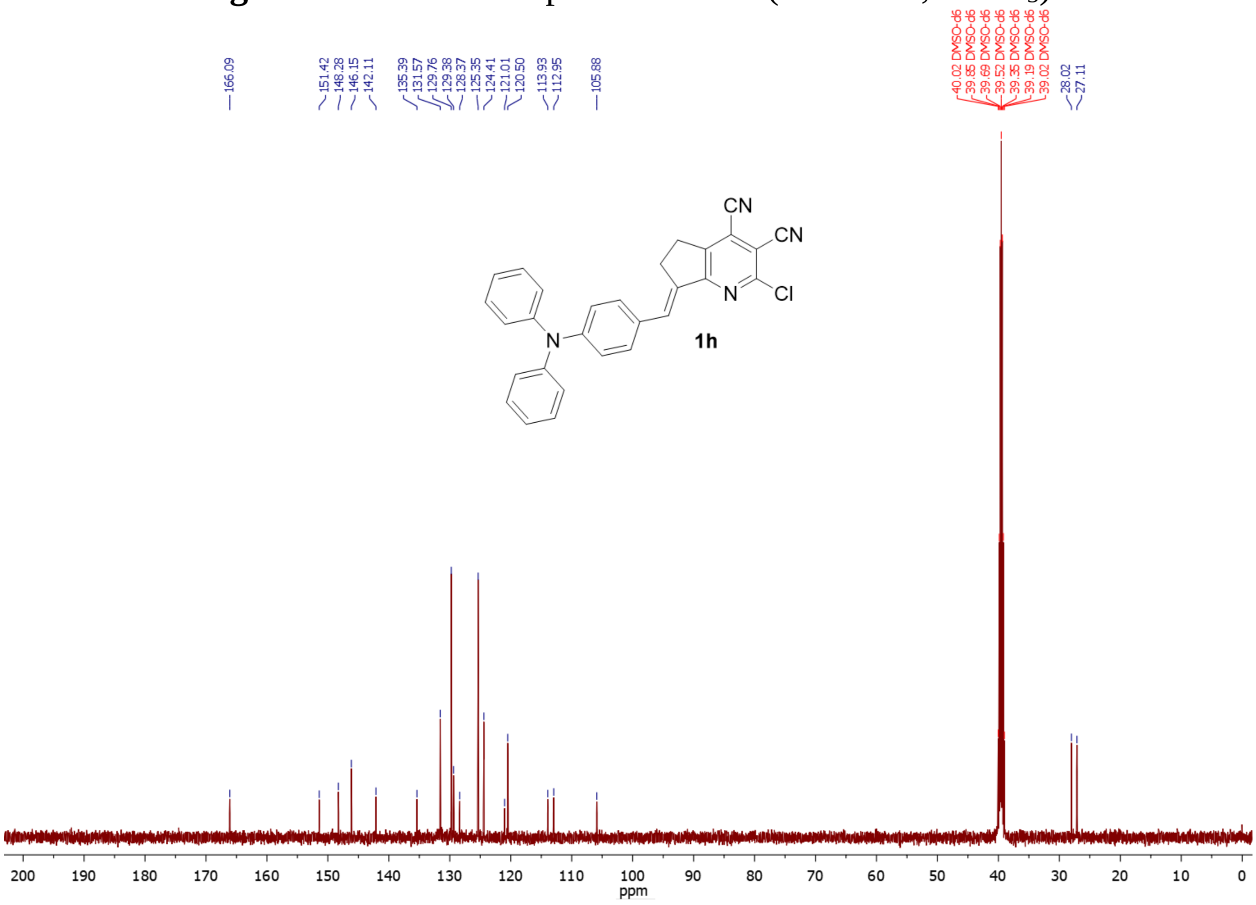

Figure S26. ^{13}C NMR spectrum of **1h** (126 MHz, DMSO-d_6).

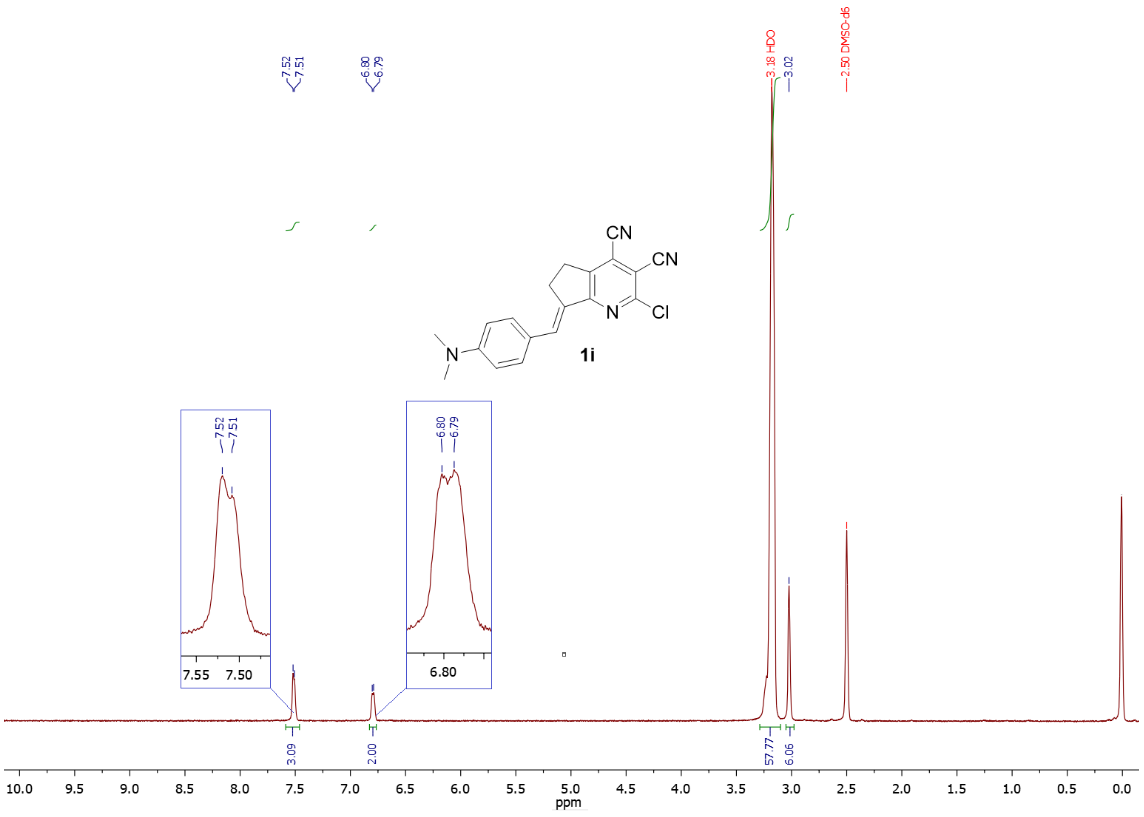

Figure S27. ^{1}H NMR spectrum of **1i** (500 MHz, DMSO-d_6).

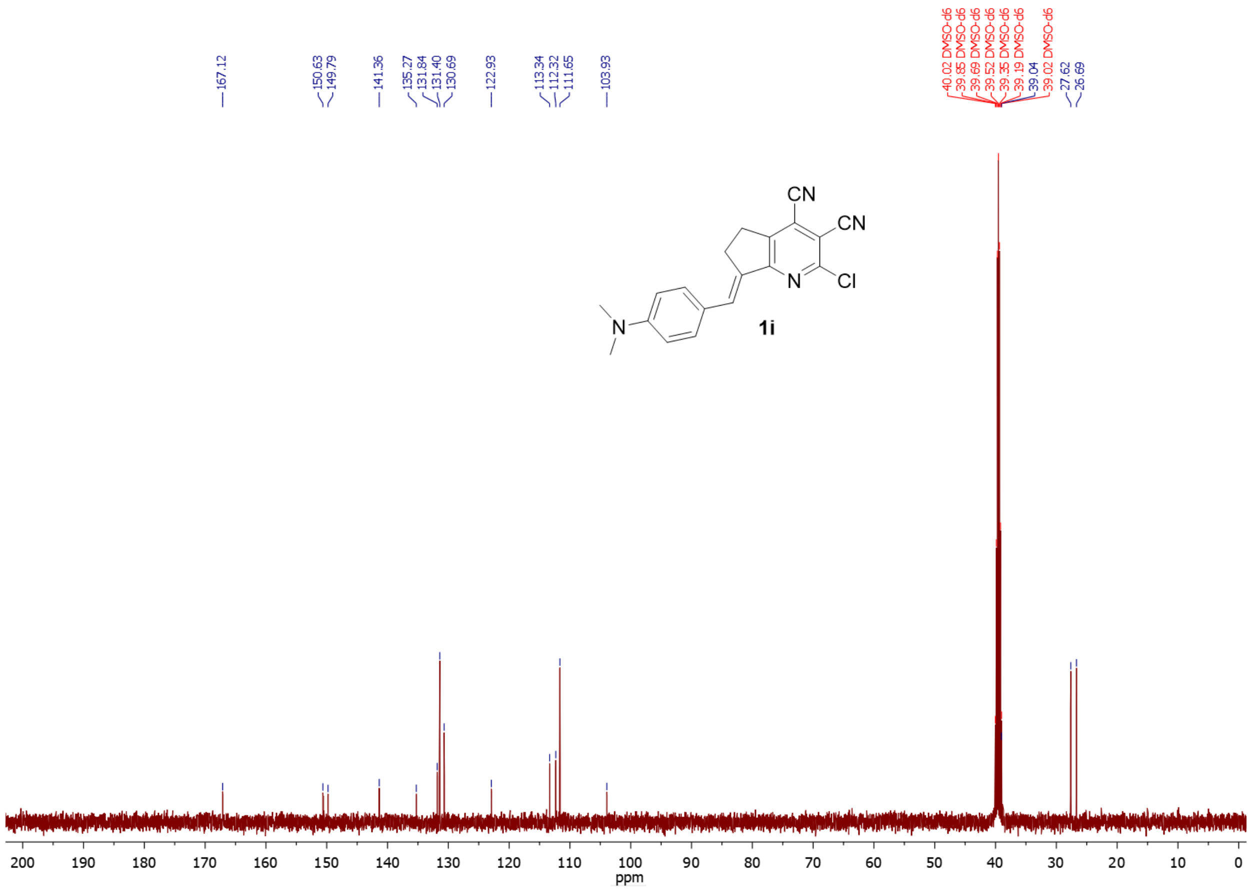

Figure S28. ^{13}C NMR spectrum of **1i** (126 MHz, DMSO-d_6).

References

1 Brouwer, A. M. *Pure Appl. Chem.* 2011, *83*, 2213–2228, doi: 10.1351/PAC-REP-10-09-31

2 Seybold, P. G.; Gouterman, M.; Callis, J. *Photochem. Photobiol.* **1969**, *9*, 229–242, doi: 10.1111/j.1751-1097.1969.tb07287.x

3 Pillai, S.; Kozlov, M.S.; Marras, A. E.; Krasnoperov, L. N.; Mustaev, A. *J. Fluoresc.* **2012**, *22*, 1021–1032, doi: 10.1007/s10895-012-1039-z

4 Ershova, A. I.; Fedoseev, S. V.; Blinov, S. A.; Ievlev, M. Y.; Lipin, K. V.; Ershov, O. V. *Org. Biomol. Chem.* **2023**, *21*, 7935–7943, doi: 10.1039/D3OB01326J
